# MRI-Based Machine Learning in Differentiation Between Benign and Malignant Breast Lesions

**DOI:** 10.3389/fonc.2021.552634

**Published:** 2021-10-18

**Authors:** Yanjie Zhao, Rong Chen, Ting Zhang, Chaoyue Chen, Muhetaer Muhelisa, Jingting Huang, Yan Xu, Xuelei Ma

**Affiliations:** ^1^ Department of Biotherapy, West China Hospital and State Key Laboratory of Biotherapy, Sichuan University, Chengdu, China; ^2^ Department of Radiology, Guiqian International General Hospital, Guiyang, China; ^3^ Department of Neurosurgery, West China Hospital, Sichuan University, Chengdu, China; ^4^ Department of Breast and Thyroid Surgery, Daping Hospital, Army Military Medical University, Chongqing, China

**Keywords:** linear discriminant analysis, differential diagnosis, machine learning, breast lesion, texture analysis, MRI

## Abstract

**Background:**

Differential diagnosis between benign and malignant breast lesions is of crucial importance relating to follow-up treatment. Recent development in texture analysis and machine learning may lead to a new solution to this problem.

**Method:**

This current study enrolled a total number of 265 patients (benign breast lesions:malignant breast lesions = 71:194) diagnosed in our hospital and received magnetic resonance imaging between January 2014 and August 2017. Patients were randomly divided into the training group and validation group (4:1), and two radiologists extracted their texture features from the contrast-enhanced T1-weighted images. We performed five different feature selection methods including Distance correlation, Gradient Boosting Decision Tree (GBDT), least absolute shrinkage and selection operator (LASSO), random forest (RF), eXtreme gradient boosting (Xgboost) and five independent classification models were built based on Linear discriminant analysis (LDA) algorithm.

**Results:**

All five models showed promising results to discriminate malignant breast lesions from benign breast lesions, and the areas under the curve (AUCs) of receiver operating characteristic (ROC) were all above 0.830 in both training and validation groups. The model with a better discriminating ability was the combination of LDA + gradient boosting decision tree (GBDT). The sensitivity, specificity, AUC, and accuracy in the training group were 0.814, 0.883, 0.922, and 0.868, respectively; LDA + random forest (RF) also suggests promising results with the AUC of 0.906 in the training group.

**Conclusion:**

The evidence of this study, while preliminary, suggested that a combination of MRI texture analysis and LDA algorithm could discriminate benign breast lesions from malignant breast lesions. Further multicenter researches in this field would be of great help in the validation of the result.

## Introduction

Breast cancer is increasingly acknowledged as a serious, worldwide public concern in women (1, 2). Several researchers have reported the incidence of breast cancer increases with age (3). This malignant and complex lesion, with a spectrum of its different subtypes, has resulted in various treatment modality, followed with heterogeneous responses and clinical outcomes (4). Early detection, diagnosis, and treatment are of crucial importance in improving the prognosis of the patients. In clinical practice, magnetic resonance imaging (MRI) is strongly suggested as the primary examination method of breast cancer for its non-ionizing radiation damage, high soft tissue resolution, and advantages in identifying the location and size of the lesions (5–8). However, a considerable problem with this kind of application is that there is hardly any competent method to separate the MRI patterns of benign breast lesions from the patterns of malignant breast lesions due to modest specificity, which usually leads to over- or undertreatment and unnecessary biopsy (9, 10). Although some studies have demonstrated that a mass size and non-mass enhancement with segmental or regional distribution indicate a breast papilloma with malignant lesions, in most cases, the differences were rather imperceptible (11). One major reason for this dilemma is the overlap between morphologic and kinetic characteristics between benign and malignant lesions (12, 13). Therefore, the importance has been raised to establish an efficient method to distinguish malignant breast lesions from benign breast lesions.

Recent development in texture analysis (TA), also known as radiomics, has led to a renewed solution to this demanding problem. TA, a mathematical method to quantify the heterogeneity in images by calculating the voxel intensity, has been applied to medical imaging (including computed tomography and MRI) and received satisfying results in the diagnosis of various lesions (14–16). Since TA can acquire the additional quantified information from the images that are not discernible to the human eye, more studies have been long established in the advantages of TA in facilitating differential diagnosis (17, 18). However, to date, limited researches have emerged to apply TA in differential diagnosis between benign and malignant breast lesions. The aim of this research has therefore been to adopt and evaluate machine learning algorithm combined with MRI TA in the discrimination of benign and malignant breast lesions.

## Materials and Methods

### Patient Selection

We retrospectively searched for patients diagnosed with benign or malignant breast lesions from January 1, 2014, to August 3, 2017, in the institution’s database. Eligibility criteria required patients to have 1) histopathological report of biopsy, 2) detailed electronic medical records, and 3) diagnostic MR scanning records before chemotherapy or surgical resection. Patients were excluded from the study if they had 1) existence of motion artifact on MR images and 2) received certain treatments (including surgical resection, chemotherapy, or radiotherapy) before MR scanning.

### MR Imaging Sequence Selection

For all the patients enrolled in this study, after laying the patient in a prone position, contrast-enhanced T1-weighted sequence was available, and the imaging was performed using a 1.5-T MR scanner with a bilateral, dedicated, 16-channel phased-array breast coil (Magnetom Aera, Siemens Medical Solutions, Germany). Dynamic series consisted of seven individual dynamic images with axial fat-suppressed T1-weighted imaging (T1WI), and the parameters were as follows: repetition time/echo time (TR/TE) = 4.62/1.75 ms, slice thickness = 1.5 mm, and space = 0 mm. Of these dynamic images, one was obtained before the intravenous injection, and six were obtained after the intravenous injection. Gadolinium-DTPA (Magnevist, Berlin, Germany) was injected as the contrast agent (0.15 mmol/kg bodyweight) at a rate of 2.0 ml/s, followed by a 15-ml saline flush.

### Image Processing and Lesion Segmentation

In this study, after a preliminary assessment on images, we selected contrast-enhanced T1-weighted (TIC) images for further analysis. Image series were imported from radiomics platform as Digital Imaging and Communications in Medicine (DICOM) files. We performed TA on LIFEx software (version 5.10, French Alternative Energies and Atomic Energy Commission) (19). We followed the guidelines of the image biomarker standardization initiative (IBSI) and manually segmented the two-dimensional region of interest (ROI) of the benign and malignant breast lesions depending on the imaging characteristic differences between the lesions and normal tissue (20). Two radiologists, blinded to the patients’ electronic medical record and histopathological diagnosis, used LIFEx software to draw the ROI with the assistance of a senior radiologist. For the purpose of higher validity as well as reproducibility, disagreements were addressed to the senior radiologist and received further discussion. The process was performed under the software protocols, and the ROI was carefully drawn layer by layer on the axial plane along the boundary of the lesions.

### Texture Feature Extraction

No specific preprocessing was conducted in the present study. The image gray-level intensity was normalized to a scale of 1 to 64 (19). Image processing steps including interpolation, re-segmentation, and discretization were automatically performed by the radiomic-specific LIFEx software. For each patients’ image series, 45 texture features recommended by the IBSI were extracted from the delineated ROIs by first order or higher order. We extracted histogram-based indices as first-order statistics. In the higher-order statistics, texture features were calculated from six matrices: gray-level co-occurrence matrix (GLCM), gray-level run length matrix (GLRLM), gray-level zone length matrix (GLZLM) (also known as gray-level size zone matrix (GLSZM)), histogram-based matrix (HISTO), neighborhood gray-level dependence matrix (NGLDM), and Shape. The main texture features for all the included patients are recorded in **Supplementary Table 1**.

### Texture Feature Selection

In **Supplementary Table 2**, we listed the definition and description of every kind of our extracted texture features. In this study, the capacity of the machine learning algorithm was limited; therefore, we could not take all the features into the analysis. Feature selection was conducted to determine the most related texture features and, additionally, avoid overfitting. Moreover, in order to find out optimal texture features, five independent selection methods were adopted, including distance correlation, gradient boosting decision tree (GBDT), least absolute shrinkage and selection operator (LASSO), random forest (RF), and extreme gradient boosting (XGBoost). These selection methods created five subsets and formed five different datasets.

### Classification

**Figure 1:**
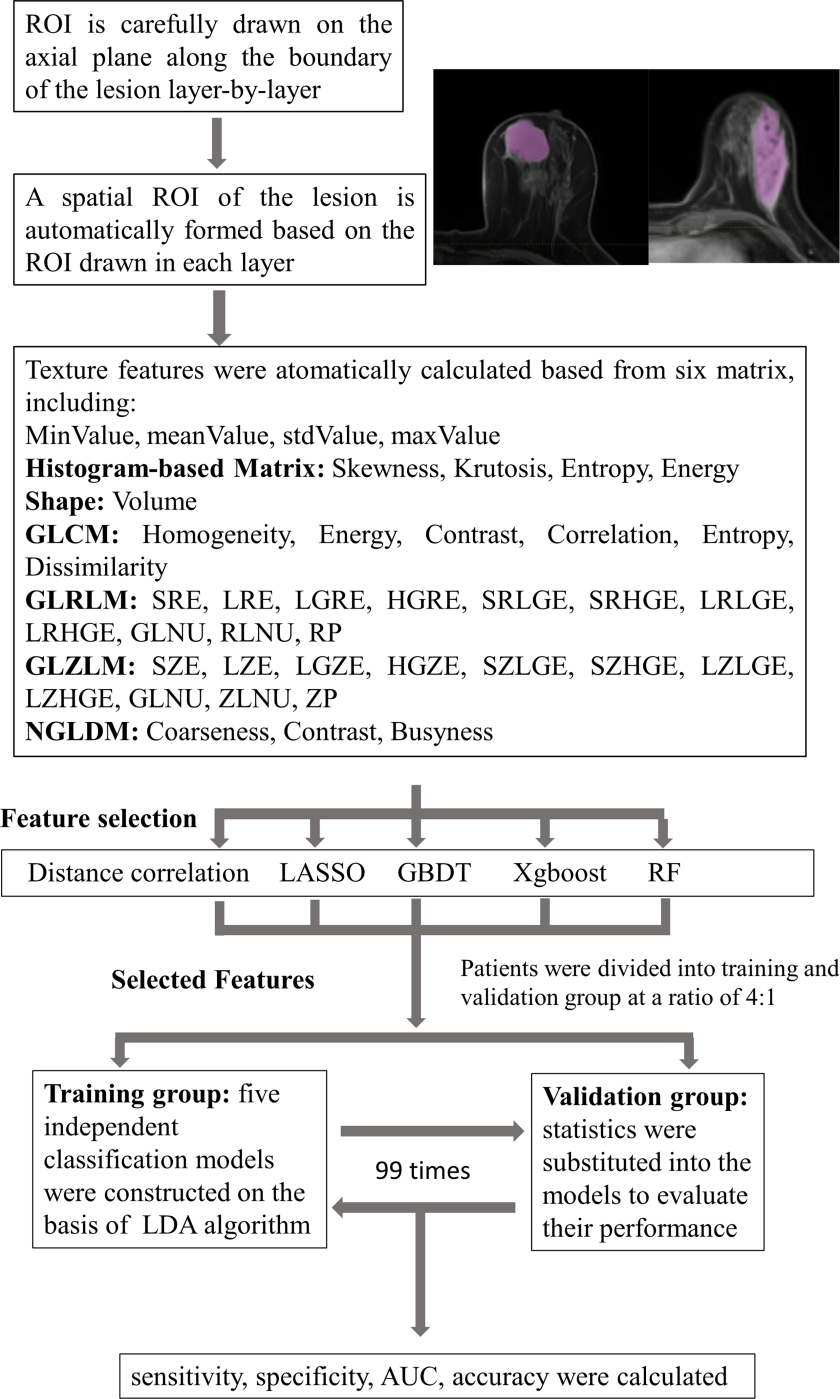
Flowchart of the MRI classification process by different selection methods. ROI, region of interest; GLCM, gray-level co-occurrence matrix; GLRLM, gray-level run length matrix; GLZLM, gray-level zone length matrix; NGLDM, neighborhood gray-level dependence matrix; LASSO, least absolute shrinkage and selection operator; GBDT, gradient boosting decision tree; RF, random forest; LDA, linear discriminant analysis; AUC, area under the receiver operating characteristic curve.

Linear discriminant analysis (LDA) is a supervised pattern recognition technique that can separate groups by searching for one or several linear combination or discriminant of predictors that maximize the ratio of between-class variance and minimize the ratio of within-class variance. Related packages were downloaded from the scikit-learn, and the models were constructed by default (21). This case study established five independent classification models on the basis of LDA algorithm: distance correlation + LDA, RF + LDA, LASSO + LDA, XGBoost + LDA, and GBDT + LDA. We randomly divided the texture features of both benign and malignant breast lesions into the training groups and validation groups (training group:validation group = 4:1). The ratio of benign and malignant breast lesions in both groups was proportional to the ratio of total benign and malignant breast lesions enrolled in our study. After training, the models were later applied with the data from the validation group, and the performance was evaluated. For each model, the randomized procedure was repeated over 100 times to appraise the robustness of the machine learning algorithm. The sensitivity, specificity, areas under the curve (AUCs), and accuracy, which represented the ability of the model to distinguish the two lesions, were later calculated in both groups. The comparison between the five different models (distance correlation + LDA, RF + LDA, LASSO + LDA, XGBoost + LDA, and GBDT + LDA) was conducted to choose a relatively suitable model with the optimal discriminative ability of benign and malignant breast lesions. The flowchart of the MRI classification of benign and malignant breast lesions in different datasets is summarized in **Figure 1**.

### Ethics Approval

Studies involving human participants were reviewed and approved by the medical ethics committee of Daping Hospital. The patients/participants’ legal guardian provided written informed consent to participate in this study.

## Results

### Patient Characteristics

**Figure 2:**
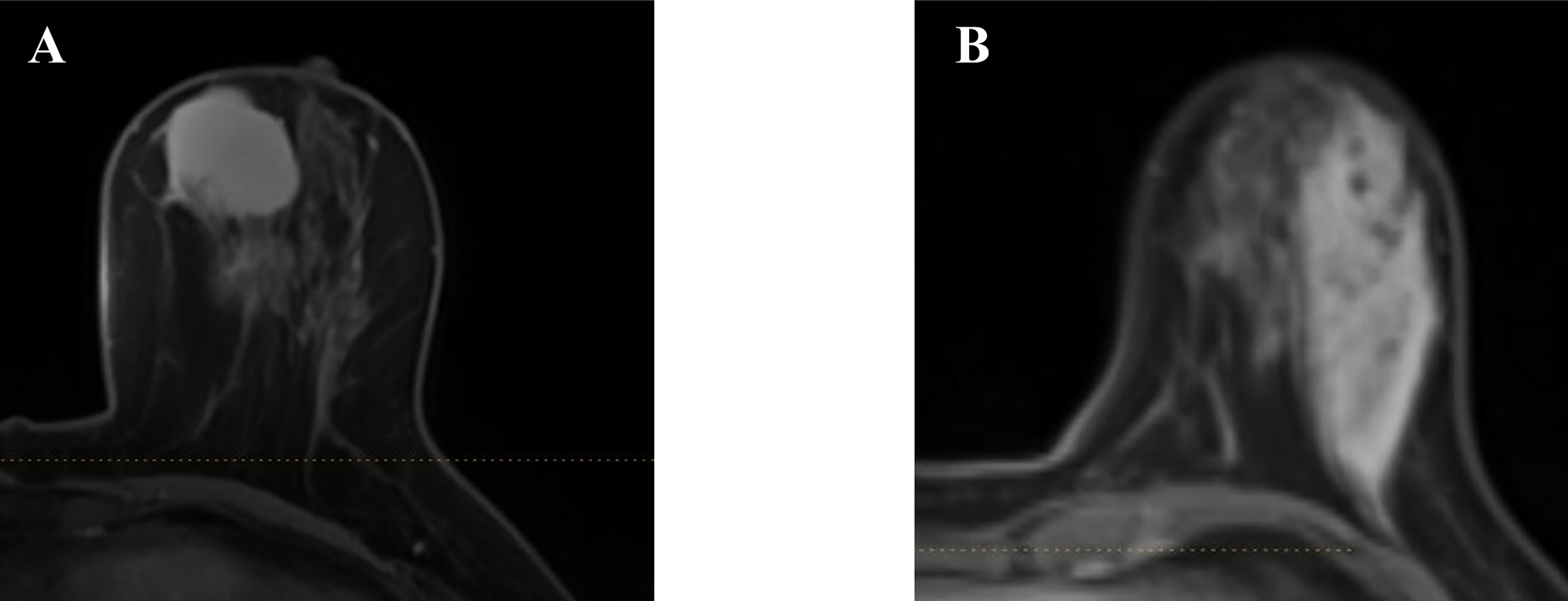
Two examples of the axial plane of contrast-enhanced T1-weighted MR images. **(A)** Malignant breast lesions. **(B)** Benign breast lesions.

A total number of 314 patients were primarily selected from the database of the institution after a retrospective review of their MR images and electronic medical record. Among them, 51 patients were excluded according to the exclusion criteria. In the remaining patients, 71 were histopathology-proven benign breast lesions, while 194 were histopathology-proven malignant breast lesions. **Table 1** displays the overview of baseline characteristics of the included patients. The median age and the age range for benign and malignant breast lesion groups were 31.9 (19–45) and 51.9 years (27–83), with a standard deviation of 6.0 and 10.3 years. In the benign lesion group, the majority comprised plasma cell mastitis (90.1%), while seven (9.9%) other patients were diagnosed with granulomatous mastitis. The numbers of malignant lesions for non-invasive carcinoma, invasive carcinoma, and others were 11 (5.7%), 194 (92.2%), and 4 (2.1%), respectively. All the patients underwent diagnostic MRI examination between January 2014 and August 2017. **Figure 2** illustrates the contrast-enhanced T1-weighted MR images of two examples (A: malignant breast lesions; B: benign breast lesions).

**Table 1:** Baseline characteristics of the 93 patients included in the analysis.

Characteristics	Benign lesions	Malignant lesions
	n = 71 (%)	n = 194 (%)
Mean age (years; SD)	31.9 ± 6	51.9 ± 10.3
Location		
Left	23 (32.4%)	89 (45.9%)
Right	43 (60.6%)	105 (54.1%)
Bilateral	5 (7.0%)	0 (0.0%)
Pathology		
PCM	64 (90.1%)	
GM	7 (9.9%)	
Non-invasive carcinoma		11 (5.7%)
Invasive carcinoma		194 (92.2%)
Others^†^		4 (2.1%)

PCM, plasma cell mastitis; GM, granulomatous mastitis.

^†^“Others” refers to carcinoma with medullary features, tubular carcinoma, invasive cribriform carcinoma, and invasive papillary carcinoma each in the present study.

### Benign *Versus* Malignant Breast Lesions

In the present study, we conducted five different texture feature selection methods on the statistics, including distance correlation, GBDT, LASSO, RF, and XGBoost. All the selected texture features are recorded and summarized in **Supplementary Table 3**. We constructed five different models based on the LDA algorithm and five different datasets of texture features. All these models had achieved high discriminant performance, and all the AUCs in these training groups are above 0.850. The detailed performance of these models (sensitivity, specificity, AUC, and accuracy) is listed in **Table 2**. The result suggested that the GBDT + LDA model achieved a statistically higher discriminative ability among the others and received the highest AUC in the training groups as well as validation groups. The sensitivity, specificity, AUC, and accuracy of the training groups’ model were 0.814, 0.779, 0.922, and 0.868, respectively; as for the validation group, the results were 0.883, 0.892, 0.911, and 0.868, respectively. The RF + LDA model also showed optimal discriminant ability with the AUC > 0.9 in the training group.

**Table 2:** The performance of five different models.

	Training				Validation			
	Sensitivity	Specificity	Accuracy	AUC	Sensitivity	Specificity	Accuracy	AUC
Distance Correlation	0.655	0.801	0.777	0.859	0.635	0.811	0.787	0.835
RF	0.753	0.863	0.839	0.906	0.675	0.865	0.825	0.881
LASSO	0.733	0.839	0.818	0.863	0.745	0.856	0.836	0.869
XGBoost	0.702	0.813	0.795	0.899	0.701	0.837	0.815	0.899
**GBDT**	**0.814**	**0.883**	**0.868**	**0.922**	**0.779**	**0.892**	**0.868**	**0.911**

LASSO, least absolute shrinkage and selection operator; GBDT, gradient boosting decision tree; RF, random forest; LDA, linear discriminant analysis; AUC, area under the receiver operating characteristic curve. We highlighted a relatively better performed model in bold values.

**Figure 3:**
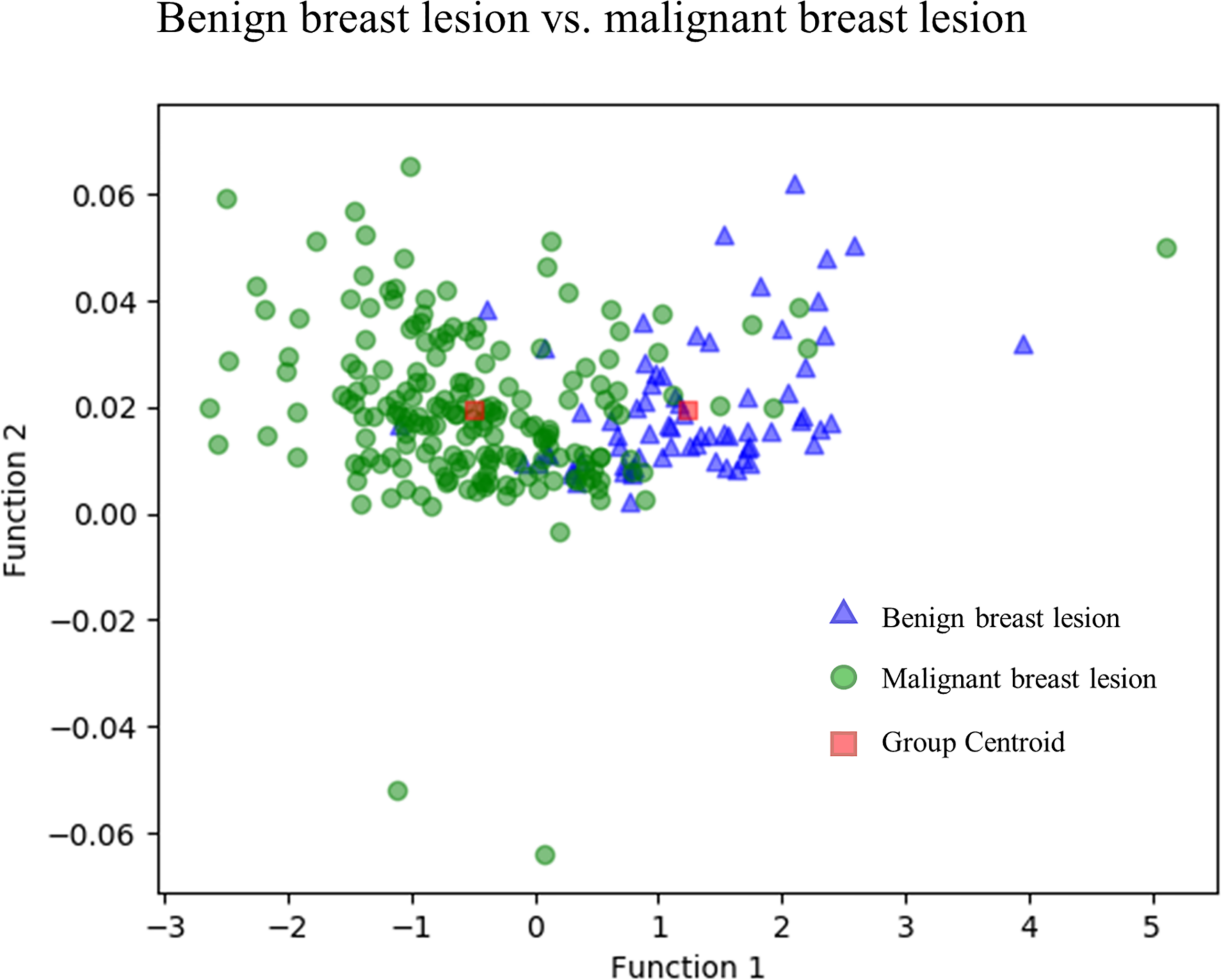
Discriminative function of the GBDT + LDA models. Distribution of the benign and malignant breast lesions that originated from multiple dimensions were reduced and reflected to a two-dimension plane. Little overlap was observed between the distribution of benign breast lesion groups (triangles) and malignant breast lesion groups (circles) and the distribution of group centroids (squares). It suggests a qualitative separation between benign and malignant breast lesions. GBDT, gradient boosting decision tree; LDA, linear discriminant analysis.

**Figure 4:**
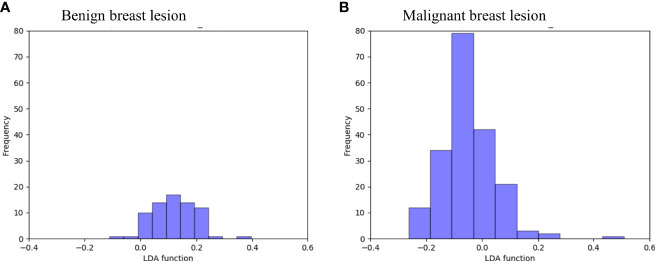
Examples of the 100 data analysis training cycles. Distribution of the direct LDA function determining benign and malignant breast lesions illustrates a promising performance of the GBDT + LDA models. GBDT, gradient boosting decision tree; LDA, linear discriminant analysis. **(A)** Distribution of the LDA function determined for the benign breast lesions for one cycle; **(B)** distribution of the LDA function determined for the malignant breast lesions for one cycle.

**Figure 3** directly illustrates the discriminative function of the GBDT + LDA models. Note that there was little overlap between the distribution of benign breast lesion groups (triangles) and malignant breast lesion groups (circles) and the distribution of group centroids (squares). The promising results indicated that GBDT + LDA models provided a qualitative separation between benign and malignant breast lesions. **Figure 4** reveals an example of 100 independent training cycles in the GBDT + LDA models, in which the findings are shown in the distribution of the direct LDA function (A: benign breast lesions; B: malignant breast lesions).

## Discussion

Prior studies had stressed the importance of distinguishing benign breast lesions from malignant breast lesions in view of redundant invasive examination and the significant differences in their treatment strategies and prognostic results (22). Traditionally, MR scanning was recommended as a sensitive modality to detect and diagnose breast lesions, together with mammography, ultrasound, and image-guided needle biopsy. However, many uncertainties still existed about the accuracy of this modality for the characteristics of benign and malignant breast lesions resemblance on the conventional MR images (12). In the current study, we examined the discriminative ability of MRI-based TA by combining five different extracted MRI-based texture feature datasets with a supervised pattern recognition technique to establish five LDA-based models. The results indicated that all these models presented good discriminant ability. Moreover, a combination of GBDT selection method for TA and LDA algorithm for classification exhibited a better performance by statistics among the others. These findings highlight the potential usefulness of machine learning to complete the separation of benign and malignant breast lesions in clinical practice.

TA was a statistical method focused on the analytic techniques and the description of image texture, which was formerly defined as the repeating patterns of local variations in gray-level intensities (23). Advances in TA had enabled recent researches to visualize spatial histologic heterogeneity, capturing image patterns that were unrecognizable to human eyes. A considerable amount of literature has been published applying TA to discriminate breast lesions. A previous study had tried to adopt a combination of texture features (GLCM entropy, GLCM Sum Average, and GLCM Homogeneity) and morphology features to diagnose benign and malignant lesions from a 2D slice of 3D images (24). This idea was further extended by researchers, and a study was conducted to investigate the utility of 3D breast lesion characterization (GLCM) by Student’s t-test in distinguishing benign and malignant lesions (25). Compared with these studies, we enrolled a larger group of texture features generated from different matrixes. Moreover, we combined machine learning to select significant texture features, and the results of our models showed a reasonably high sensitivity, specificity, AUC, and accuracy. In recent years, other studies had tried to relate breast lesions TA of contrast-enhanced MR images to the underlying lesion subtypes and received satisfying results (26).

The past decade had seen the rapid development of machine learning applied to MR images in different fields (27–29). After evaluation of the performance of the selection methods and classification algorithms, predictive models were created for tumor grading, diagnosis of interest, and clinical outcome. The association between molecular expression (Ki67 and HER2) and contrast-enhanced MRI features were also observed in some studies (30, 31). Other researchers investigated machine learning in different radiological techniques such as mammograms and ultrasound (32, 33). Previous research had indicated that integration of 10-fold cross-validation method and machine learning into the interpretation of MR images can help to make decisive rules to manage suspicious breast lesions (34). Another study also achieved promising diagnostic results in discriminating breast lesions with deep learning method using ResNet50 (35). In our study, we built five different classification models based on LDA algorithm, and a relatively optimal machine learning model with a combination of LDA + GBDT demonstrated a non-inferior accuracy of 0.868 and 0.892 in the training and validation groups, respectively. The current findings to evaluate the performance of five different feature selection methods combined with LDA algorithms add to a growing body of literature on computer-aided diagnosis of breast lesions.

Many studies in the medical image field of machine learning algorithm had noticed the influence on the diagnostic performance caused by the adoption of different texture features. Recently, the IBSI had standardized the extraction of image biomarkers from imaging to present a high-throughput quantitative image analysis. All the texture features included in this study were recommended in the IBSI feature reference values and added to the quality of our research (20). Previous researches had discussed various kinds of selection methods including Student’s t-test, Mann–Whitney U test, ReliefF algorithm (36, 37). Based on the results, the interference of the selection method cannot be ruled out. Compared with previous studies, we extracted a relatively large number of parameters from different matrices, which increased the possibility to select the optimal features. Moreover, in order to establish the optimal classifier, we chose five different feature selection methods (distance correlation, RF, LASSO, XGBoost, and GBDT) with which the performances were later evaluated. Overall, the result of this study indicates that all the models showed good performance and that the GBDT + LDA model achieved a better performance for benign breast lesions from malignant breast lesions with the highest AUC of 0.922 in the training group. GBDT was proposed as a tree-based algorithm based on a greedy strategy (called gradient boosting) that evaluates the importance of a texture feature through the time it used as branching point for the tree. However, the results of this study must be interpreted with caution because no significant differences were observed between the performance of all the models, and the variance in AUC may result from the statistical group. Therefore, the results can only be suggested as a hypothesis generation and required verification from future, larger studies.

Several limitations to this pilot study need to be acknowledged. First, this study is single-centered. The selection bias for patients is unavoidable and may have influenced the analysis. Second, the sample size was relatively small, and a greater size of the sample is expected for further study to validate the results. Third, the results obtained in this study did not receive external validation in other datasets, and the diagnostic ability of this model may be influenced due to different MR scanners and image processing procedure. Fourth, only texture features from contrast-enhanced T1-weighted (TIC) images were introduced in this study. Further studies are required to explore the classifier adapted with texture features from other sequences.

## Conclusion

The study was undertaken to design optimal classification model using texture features combined with machine learning algorithm and evaluate its sensitivity, specificity, AUC, and accuracy. We used five selection methods and established five discriminative models, and their performances were evaluated. In general, therefore, it seems that texture features have potential to be utilized in discriminating benign breast lesions from malignant breast lesions. More broadly, future multicenter researches with more patients in this field would be of great help to validate this preliminary result.

## Data Availability Statement

The datasets presented in this study can be found in online repositories. The names of the repository/repositories and accession number(s) can be found in https://scikit-learn.org/stable/supervised_learning.html#supervised-learning.

## Ethics Statement

Studies involving human participants were reviewed and approved by medical ethics committee of Daping Hospital. The patients/participants’ legal guardian provided written informed consent to participate in this study.

## Author Contributions

YZ extracted the texture features from MR images and wrote the manuscript. RC contributed equally to this paper and extracted the texture features from MR images. CC, MM, TZ, and JH conducted all the analysis in this paper. XM is the first corresponding author of this paper and proposed the topic and methods of this paper. YX is the second corresponding author of this paper and provides the detailed information as well as the MR images of the patients. All authors contributed to the article and approved the submitted version.

## Funding

This work was supported by the National Natural Science Foundation of China under Grant No. 81472482; and the Clinical Technology Innovation and Cultivation Project of Army Military Medical University of China under Grant No. CX2019LC120.

## Conflict of Interest

The authors declare that the research was conducted in the absence of any commercial or financial relationships that could be construed as a potential conflict of interest.

## Publisher’s Note

All claims expressed in this article are solely those of the authors and do not necessarily represent those of their affiliated organizations, or those of the publisher, the editors and the reviewers. Any product that may be evaluated in this article, or claim that may be made by its manufacturer, is not guaranteed or endorsed by the publisher.
