# Supplementary material for: MRI-Based Machine Learning in Differentiation Between Benign and Malignant Breast Lesions
Source: Front Oncol. 2021 Oct 18;11:552634. doi: 10.3389/fonc.2021.552634 (PMC8558475; doi:10.3389/fonc.2021.552634)
Supplement: Supplementary file 1 [file Table_1.docx]

|  |
| --- |
|  |

Supplement material 1: Definition and description of texture parameters

| Matrixes | Defination | TA feature | Description |  |
| --- | --- | --- | --- | --- |
|  |  | Contrast | Local variations in the GLCM |  |
|  |  |  |  |  |
|  | The arrangements of | Correlation | Linear dependency of grey-levels in GLCM |  |
|  |  |  |  |  |
|  |  | Dissimilarity | Variation of grey-level voxel pairs |  |
| Co-occurrence | pairs of voxels to |  |  |  |
| matrix (GLCM) | extract textural | Energy | Uniformity of grey-level voxel pairs. |  |
|  | indices |  |  |  |
|  |  | Entropy | Randomness of grey-level voxel pairs |  |
|  |  |  |  |  |
|  |  |  |  |  |
|  |  | Homogeneity | Homogeneity of grey-level voxel pairs |  |
|  |  | SRE | Distribution of the short homogeneous runs in an image |  |
|  |  |  |  |  |
|  |  | LRE | Distribution of the long homogeneous runs in an image |  |
|  |  |  |  |  |
|  |  | LGRE | Distribution of the low grey-level runs |  |
|  |  |  |  |  |
|  |  | HGRE | Distribution of the high grey-level runs |  |
|  | The size of |  |  |  |
| Grey-Level Run |  | SRLGE | Distribution of the short homogenous runs with low grey-levels |  |
|  | homogenous grey- |  |  |  |
| Length Matrix |  | SRHGE | Distribution of the short homogenous runs with high grey-levels |  |
|  | level runs for each |  |  |  |
| (GLRLM) |  |  |  |  |
|  |  | LRLGE | Distribution of the long homogeneous runs with low grey-levels |  |
|  | grey level |  |  |  |
|  |  |  |  |  |
|  |  | LRHGE | Distribution of the long homogeneous runs with high grey-levels |  |
|  |  |  |  |  |
|  |  | GLNUr | Non-uniformity of the grey-levels of the homogeneous runs |  |
|  |  |  |  |  |
|  |  | RLNU | Length of the homogeneous runs |  |
|  |  |  |  |  |
|  |  | RP | Homogeneity of the homogeneous runs |  |
|  |  | SZE | Distribution of the short homogeneous zones in an image |  |
|  |  |  |  |  |
|  |  | LZE | Distribution of the long homogeneous zones in an image |  |
|  |  |  |  |  |
|  |  | LGZE | Distribution of the low grey-level zones |  |
|  |  |  |  |  |
|  |  | HGZE | Distribution of the high grey-level zones |  |
|  |  |  |  |  |
|  | The information on | SZLGE | Distribution of the short homogenous zones with low grey-levels |  |
|  |  |  |  |  |
|  |  | SZHGE | Distribution of the short homogenous zones with high grey-levels |  |
| Grey-Level Zone | the size of |  |  |  |
|  |  |  |  |  |
| Length Matrix | homogenous zones | LZLGE | Distribution of the long homogenous zones with low grey-levels |  |
| (GLZLM) | for each grey-level |  |  |  |
|  |  | LZHGE | Distribution of the long homogenous zones with high grey-levels |  |
|  | in three dimensions |  |  |  |
|  |  |  |  |  |
|  |  | GLNUz | Non-uniformity of the grey-levels of the homogeneous zones |  |
|  |  |  |  |  |
|  |  |  |  |  |
|  |  | RLNU | Length of the homogeneous runs |  |
|  |  |  |  |  |
|  |  | ZP | Homogeneity of the homogeneous zones |  |
|  |  |  |  |  |
|  |  | Sphericity | Measures how spherical a volume of interest is |  |
|  |  |  |  |  |
|  |  | Compacity | Measures the degree to which the volume of interest is compact |  |
|  |  | Skewness | Measures the asymmetry of the grey-level distribution in the histogram. |  |
|  |  |  |  |  |
|  | The information | Kurtosis | Measures whether the grey-level distribution is peaked or flat relative to a |  |
| Histogram | derived from global |  | normal distribution |  |
|  |  |  |  |  |
|  | histogram analysis | Entropy | Measures the randomness of the distribution |  |
|  |  |  |  |  |
|  |  | Energy | Measures the uniformity of the distribution |  |
| Neighbourhood | The difference of | Coarseness | Level of spatial rate of change in intensity |  |
|  | grey-level between |  |  |  |
| Grey-Level |  | Contrast | Intensity difference between neighbouring regions |  |
|  | one voxel and its 26 |  |  |  |
| Different Matrix |  |  |  |  |
|  |  |  |  |  |
|  | neighbourhoods in | Busyness | Spatial frequency of changes in intensity |  |
| (NGLDM) |  |  |  |  |
|  | three dimensions |  |  |  |
|  |  |  |  |  |
| Shape |  | Volume | Volume of Interest in mL and in voxels. |  |
