# Supplementary material for: MRI-Based Machine Learning in Differentiation Between Benign and Malignant Breast Lesions
Source: Front Oncol. 2021 Oct 18;11:552634. doi: 10.3389/fonc.2021.552634 (PMC8558475; doi:10.3389/fonc.2021.552634)
Supplement: Supplementary file 2 [file Table_2.docx]

| Patient | 1 | 2 | 3 | 4 | 5 | 6 | 7 | 8 | 9 | 10 | 11 |
| --- | --- | --- | --- | --- | --- | --- | --- | --- | --- | --- | --- |
| Lesion (benign=0,malignant=1) | 0 | 0 | 0 | 0 | 0 | 0 | 0 | 0 | 0 | 0 | 0 |
| minValue | 199 | 99 | 41 | 69 | 294 | 102 | 95 | 197 | 150 | 56 | 54 |
| meanValue | 396 | 225 | 96.7 | 132 | 376 | 454 | 186 | 438 | 435 | 98.6 | 319 |
| stdValue | 57.7 | 39.2 | 17 | 13.7 | 19.2 | 50.2 | 13.7 | 62 | 87.5 | 9.97 | 64.8 |
| maxValue | 541 | 301 | 143 | 158 | 439 | 560 | 217 | 554 | 592 | 119 | 499 |
| HISTO_Skewness | -0.296 | -0.729 | -0.0466 | -1.34 | -0.69 | -2.4 | -1.59 | -0.877 | -0.799 | -1.46 | -0.683 |
| HISTO_Kurtosis | 2.91 | 3.21 | 2.52 | 5.29 | 4.72 | 12.2 | 8.78 | 3.42 | 3.29 | 6.31 | 3.43 |
| HISTO_Entropy_log2 | 5.37 | 5.54 | 5.33 | 5.01 | 4.95 | 4.43 | 4.67 | 5.29 | 5.56 | 4.97 | 5.19 |
| GLCM_Homogeneity | 0.166 | 0.194 | 0.207 | 0.234 | 0.24 | 0.31 | 0.297 | 0.216 | 0.199 | 0.237 | 0.217 |
| GLCM_Energy | 0.00442 | 0.00171 | 0.00217 | 0.00729 | 0.00441 | 0.00578 | 0.00392 | 0.00461 | 0.00122 | 0.0124 | 0.00142 |
| GLCM_Contrast | 189 | 265 | 118 | 155 | 73.1 | 63.7 | 54.7 | 156 | 204 | 113 | 125 |
| GLCM_Correlation | 0.222 | 0.272 | 0.388 | 0.0819 | 0.279 | 0.45 | 0.47 | 0.141 | 0.379 | 0.31 | 0.263 |
| GLCM_Entropy_log2 | 8.04 | 9.47 | 9.1 | 7.5 | 8.12 | 8.19 | 8.65 | 8.07 | 10 | 6.67 | 9.94 |
| GLCM_Dissimilarity | 11.1 | 11.9 | 8.43 | 8.54 | 6.5 | 5.17 | 5.11 | 9.11 | 10.5 | 7.15 | 8.28 |
| GLRLM_SRE | 0.984 | 0.976 | 0.978 | 0.982 | 0.977 | 0.956 | 0.955 | 0.981 | 0.977 | 0.985 | 0.975 |
| GLRLM_LRE | 1.07 | 1.11 | 1.09 | 1.08 | 1.1 | 1.22 | 1.21 | 1.09 | 1.1 | 1.06 | 1.11 |
| GLRLM_LGRE | 0.00314 | 0.00349 | 0.00224 | 0.00239 | 0.00317 | 0.00113 | 0.00106 | 0.00268 | 0.0027 | 0.00634 | 0.00144 |
| GLRLM_HGRE | 1510 | 1780 | 1370 | 2210 | 1430 | 2510 | 2350 | 2020 | 1900 | 1990 | 1570 |
| GLRLM_SRLGE | 0.00313 | 0.00343 | 0.00221 | 0.00238 | 0.00315 | 0.00111 | 0.00103 | 0.00267 | 0.00266 | 0.00634 | 0.00142 |
| GLRLM_SRHGE | 1490 | 1730 | 1340 | 2170 | 1400 | 2390 | 2240 | 1980 | 1850 | 1960 | 1530 |
| GLRLM_LRLGE | 0.00321 | 0.00377 | 0.00234 | 0.00243 | 0.00325 | 0.00123 | 0.00116 | 0.00273 | 0.00289 | 0.00637 | 0.00153 |
| GLRLM_LRHGE | 1620 | 2010 | 1500 | 2410 | 1580 | 3080 | 2860 | 2230 | 2120 | 2120 | 1760 |
| GLRLM_GLNU | 14.7 | 35.9 | 28.1 | 24 | 19.3 | 121 | 135 | 20.4 | 55.6 | 9.05 | 228 |
| GLRLM_RLNU | 506 | 1330 | 927 | 559 | 458 | 1740 | 2420 | 637 | 2130 | 219 | 6640 |
| GLRLM_RP | 0.979 | 0.968 | 0.971 | 0.976 | 0.969 | 0.94 | 0.94 | 0.974 | 0.97 | 0.981 | 0.966 |
| NGLDM_Coarseness | 0.016 | 0.0052 | 0.00777 | 0.0131 | 0.0145 | 0.0034 | 0.00264 | 0.0129 | 0.00327 | 0.0302 | 0.000991 |
| NGLDM_Contrast | 0.359 | 0.472 | 0.352 | 0.259 | 0.246 | 0.0918 | 0.0955 | 0.348 | 0.484 | 0.345 | 0.236 |
| NGLDM_Busyness | 0.0362 | 0.0716 | 0.078 | 0.0305 | 0.0414 | 0.0839 | 0.108 | 0.0342 | 0.105 | 0.0204 | 0.351 |
| GLZLM_SZE | 0.815 | 0.769 | 0.777 | 0.851 | 0.781 | 0.68 | 0.682 | 0.806 | 0.786 | 0.822 | 0.754 |
| GLZLM_LZE | 2.37 | 3.46 | 3.42 | 3.04 | 4.56 | 19.4 | 20 | 2.97 | 3.42 | 2.06 | 4.15 |
| GLZLM_LGZE | 0.00381 | 0.00365 | 0.00277 | 0.003 | 0.00422 | 0.00178 | 0.00163 | 0.00342 | 0.00304 | 0.00793 | 0.00176 |

Supplementary table 2: Detailed statistics of the extracted texture features for every patient

| Patient | 12 | 13 | 14 | 15 | 16 | 17 | 18 | 19 | 20 | 21 | 22 |
| --- | --- | --- | --- | --- | --- | --- | --- | --- | --- | --- | --- |
| Lesion (benign=0,malignant=1) | 0 | 0 | 0 | 0 | 0 | 0 | 0 | 0 | 0 | 0 | 0 |
| minValue | 236 | 89 | 53 | 27 | 117 | 205 | 231 | 354 | 60 | 151 | 72 |
| meanValue | 470 | 313 | 109 | 110 | 230 | 475 | 416 | 458 | 349 | 371 | 275 |
| stdValue | 63.4 | 97.5 | 17.1 | 15.2 | 27.4 | 59.8 | 52.7 | 38.7 | 38.3 | 40.1 | 38.2 |
| maxValue | 586 | 590 | 142 | 148 | 297 | 609 | 497 | 540 | 406 | 503 | 375 |
| HISTO_Skewness | -1.1 | -0.257 | -0.818 | -0.754 | -0.716 | -1.05 | -1.22 | -0.283 | -2.68 | -1.38 | -0.927 |
| HISTO_Kurtosis | 3.85 | 2.5 | 3.67 | 4.42 | 3.67 | 5.35 | 4.05 | 2.5 | 13.7 | 6.44 | 5.36 |
| HISTO_Entropy_log2 | 5.33 | 5.58 | 5.31 | 4.95 | 5.23 | 5.09 | 5.26 | 5.44 | 4.32 | 4.66 | 4.89 |
| GLCM_Homogeneity | 0.243 | 0.174 | 0.167 | 0.22 | 0.226 | 0.22 | 0.24 | 0.207 | 0.34 | 0.284 | 0.338 |
| GLCM_Energy | 0.0026 | 0.00136 | 0.00487 | 0.0023 | 0.00161 | 0.00417 | 0.0145 | 0.00854 | 0.00727 | 0.00432 | 0.00557 |
| GLCM_Contrast | 77.6 | 219 | 295 | 101 | 106 | 110 | 72.5 | 173 | 63.5 | 64 | 49 |
| GLCM_Correlation | 0.614 | 0.258 | 0.184 | 0.242 | 0.435 | 0.128 | 0.396 | 0.472 | 0.351 | 0.314 | 0.424 |
| GLCM_Entropy_log2 | 8.91 | 9.73 | 7.85 | 9.13 | 9.75 | 8.14 | 6.52 | 7.05 | 7.85 | 8.45 | 8.04 |
| GLCM_Dissimilarity | 6.66 | 11.5 | 12.7 | 7.6 | 7.72 | 7.82 | 6.42 | 9.62 | 4.82 | 5.72 | 4.48 |
| GLRLM_SRE | 0.976 | 0.981 | 0.984 | 0.979 | 0.971 | 0.98 | 0.987 | 0.982 | 0.945 | 0.961 | 0.961 |
| GLRLM_LRE | 1.11 | 1.08 | 1.07 | 1.09 | 1.13 | 1.09 | 1.06 | 1.08 | 1.26 | 1.19 | 1.19 |
| GLRLM_LGRE | 0.00215 | 0.0128 | 0.00554 | 0.00122 | 0.00159 | 0.00264 | 0.00496 | 0.00749 | 0.00113 | 0.00142 | 0.00134 |
| GLRLM_HGRE | 2000 | 1000 | 1790 | 2050 | 1760 | 1960 | 2170 | 1500 | 2940 | 1690 | 1940 |
| GLRLM_SRLGE | 0.00213 | 0.0119 | 0.00553 | 0.00121 | 0.00157 | 0.00262 | 0.00496 | 0.00748 | 0.00111 | 0.0014 | 0.00132 |
| GLRLM_SRHGE | 1940 | 985 | 1750 | 2010 | 1700 | 1920 | 2140 | 1470 | 2760 | 1620 | 1860 |
| GLRLM_LRLGE | 0.00222 | 0.0189 | 0.00558 | 0.00127 | 0.00167 | 0.00268 | 0.005 | 0.00755 | 0.00121 | 0.00153 | 0.00144 |
| GLRLM_LRHGE | 2250 | 1080 | 1930 | 2250 | 2010 | 2150 | 2320 | 1640 | 3780 | 2030 | 2340 |
| GLRLM_GLNU | 35.3 | 27.9 | 9.9 | 62.7 | 103 | 24.4 | 8.39 | 4.94 | 100 | 83.5 | 69.1 |
| GLRLM_RLNU | 1090 | 1140 | 297 | 1520 | 2980 | 637 | 252 | 178 | 1320 | 1440 | 1430 |
| GLRLM_RP | 0.968 | 0.975 | 0.979 | 0.972 | 0.961 | 0.973 | 0.983 | 0.977 | 0.926 | 0.948 | 0.948 |
| NGLDM_Coarseness | 0.00898 | 0.00529 | 0.0177 | 0.00414 | 0.00225 | 0.013 | 0.0325 | 0.0436 | 0.00383 | 0.0046 | 0.00705 |
| NGLDM_Contrast | 0.242 | 0.628 | 0.59 | 0.221 | 0.247 | 0.206 | 0.385 | 0.646 | 0.109 | 0.115 | 0.0874 |
| NGLDM_Busyness | 0.0352 | 0.111 | 0.0232 | 0.113 | 0.159 | 0.0311 | 0.0136 | 0.0166 | 0.0738 | 0.0797 | 0.0487 |
| GLZLM_SZE | 0.786 | 0.81 | 0.825 | 0.779 | 0.738 | 0.774 | 0.866 | 0.821 | 0.703 | 0.711 | 0.721 |
| GLZLM_LZE | 3.84 | 2.87 | 2.46 | 3.1 | 5.02 | 2.79 | 2 | 2.56 | 30.3 | 8.26 | 9.47 |
| GLZLM_LGZE | 0.00281 | 0.00581 | 0.00704 | 0.00151 | 0.00211 | 0.00349 | 0.00593 | 0.00958 | 0.0021 | 0.00206 | 0.00198 |

| Patient | 23 | 24 | 25 | 26 | 27 | 28 | 29 | 30 | 31 | 32 | 33 |
| --- | --- | --- | --- | --- | --- | --- | --- | --- | --- | --- | --- |
| Lesion (benign=0,malignant=1) | 0 | 0 | 0 | 0 | 0 | 0 | 0 | 0 | 0 | 0 | 0 |
| minValue | 74 | 96 | 193 | 162 | 94 | 70 | 98 | 46 | 62 | 289 | 75 |
| meanValue | 124 | 227 | 391 | 570 | 251 | 203 | 253 | 92.7 | 193 | 485 | 122 |
| stdValue | 17 | 17.3 | 40.8 | 103 | 41 | 29.8 | 35.7 | 10.5 | 26.3 | 41.8 | 12.4 |
| maxValue | 169 | 280 | 484 | 729 | 366 | 264 | 328 | 116 | 243 | 580 | 150 |
| HISTO_Skewness | -0.199 | -2.48 | -1.15 | -1.25 | -0.724 | -1.28 | -1.04 | -1.2 | -1.25 | -0.879 | -0.636 |
| HISTO_Kurtosis | 2.65 | 15.4 | 5.44 | 4.42 | 4.11 | 5.12 | 4.43 | 4.66 | 5.12 | 3.68 | 3.1 |
| HISTO_Entropy_log2 | 5.44 | 4.22 | 5.04 | 5.2 | 5.18 | 5.08 | 5.18 | 5.03 | 5.03 | 5.1 | 5.25 |
| GLCM_Homogeneity | 0.176 | 0.345 | 0.273 | 0.19 | 0.224 | 0.228 | 0.219 | 0.233 | 0.251 | 0.257 | 0.232 |
| GLCM_Energy | 0.00167 | 0.00743 | 0.00281 | 0.00478 | 0.00731 | 0.00389 | 0.00253 | 0.0024 | 0.00312 | 0.00224 | 0.00297 |
| GLCM_Contrast | 208 | 40.3 | 88.9 | 298 | 99.2 | 144 | 123 | 104 | 105 | 80.5 | 120 |
| GLCM_Correlation | 0.264 | 0.267 | 0.426 | 0.247 | 0.315 | 0.211 | 0.328 | 0.425 | 0.39 | 0.513 | 0.377 |
| GLCM_Entropy_log2 | 9.49 | 7.74 | 8.84 | 7.89 | 7.47 | 8.28 | 8.93 | 9.25 | 8.77 | 9.28 | 8.74 |
| GLCM_Dissimilarity | 11.2 | 4.09 | 6.34 | 12.6 | 7.67 | 8.41 | 8.09 | 7.54 | 7.24 | 6.48 | 7.96 |
| GLRLM_SRE | 0.981 | 0.946 | 0.965 | 0.979 | 0.983 | 0.976 | 0.977 | 0.972 | 0.972 | 0.969 | 0.972 |
| GLRLM_LRE | 1.08 | 1.25 | 1.16 | 1.09 | 1.08 | 1.11 | 1.1 | 1.13 | 1.13 | 1.14 | 1.12 |
| GLRLM_LGRE | 0.00268 | 0.0011 | 0.00176 | 0.00328 | 0.00322 | 0.00251 | 0.00198 | 0.00229 | 0.00191 | 0.00114 | 0.00267 |
| GLRLM_HGRE | 1300 | 2170 | 2020 | 2290 | 1490 | 2060 | 2000 | 1950 | 2260 | 1980 | 1720 |
| GLRLM_SRLGE | 0.00265 | 0.00107 | 0.00174 | 0.00327 | 0.00321 | 0.0025 | 0.00196 | 0.00224 | 0.00186 | 0.00112 | 0.00265 |
| GLRLM_SRHGE | 1280 | 2050 | 1940 | 2240 | 1460 | 2000 | 1950 | 1890 | 2200 | 1910 | 1670 |
| GLRLM_LRLGE | 0.00278 | 0.00121 | 0.00184 | 0.00333 | 0.00328 | 0.00258 | 0.00203 | 0.00248 | 0.0021 | 0.00121 | 0.00275 |
| GLRLM_LRHGE | 1420 | 2720 | 2360 | 2540 | 1630 | 2300 | 2210 | 2220 | 2590 | 2280 | 1960 |
| GLRLM_GLNU | 33.2 | 140 | 39.3 | 13.1 | 19.7 | 21.5 | 29.3 | 80.7 | 63.8 | 77 | 22.4 |
| GLRLM_RLNU | 1160 | 1700 | 994 | 372 | 555 | 551 | 827 | 1910 | 1570 | 2040 | 655 |
| GLRLM_RP | 0.975 | 0.929 | 0.954 | 0.973 | 0.977 | 0.968 | 0.97 | 0.962 | 0.963 | 0.959 | 0.963 |
| NGLDM_Coarseness | 0.00505 | 0.00336 | 0.0077 | 0.0147 | 0.0161 | 0.0127 | 0.00817 | 0.00329 | 0.00507 | 0.00374 | 0.0103 |
| NGLDM_Contrast | 0.504 | 0.0551 | 0.18 | 0.517 | 0.215 | 0.289 | 0.29 | 0.205 | 0.188 | 0.203 | 0.339 |
| NGLDM_Busyness | 0.101 | 0.0804 | 0.0479 | 0.0299 | 0.0283 | 0.0314 | 0.047 | 0.0838 | 0.0671 | 0.102 | 0.0455 |
| GLZLM_SZE | 0.817 | 0.697 | 0.718 | 0.821 | 0.839 | 0.775 | 0.789 | 0.76 | 0.756 | 0.742 | 0.781 |
| GLZLM_LZE | 2.69 | 26.8 | 6.33 | 2.84 | 2.65 | 3.77 | 3.38 | 5.28 | 5 | 5.99 | 4.63 |
| GLZLM_LGZE | 0.0031 | 0.0019 | 0.00264 | 0.00424 | 0.00401 | 0.00339 | 0.00258 | 0.00252 | 0.00186 | 0.0015 | 0.0037 |

| Patient | 34 | 35 | 36 | 37 | 38 | 39 | 40 | 41 | 42 | 43 | 44 |
| --- | --- | --- | --- | --- | --- | --- | --- | --- | --- | --- | --- |
| Lesion (benign=0,malignant=1) | 0 | 0 | 0 | 0 | 0 | 0 | 0 | 0 | 0 | 0 | 0 |
| minValue | 340 | 351 | 96 | 344 | 51 | 144 | 165 | 74 | 63 | 128 | 200 |
| meanValue | 485 | 427 | 135 | 540 | 209 | 267 | 281 | 176 | 117 | 238 | 416 |
| stdValue | 57.2 | 25.3 | 17.3 | 48.4 | 58.5 | 22.3 | 31 | 21.7 | 16.8 | 29.5 | 26.8 |
| maxValue | 609 | 499 | 175 | 641 | 308 | 306 | 334 | 241 | 175 | 316 | 463 |
| HISTO_Skewness | -0.457 | 0.184 | -0.235 | -1.06 | -0.658 | -1.93 | -1.37 | -1.6 | 0.14 | -0.739 | -2.74 |
| HISTO_Kurtosis | 2.46 | 2.86 | 2.82 | 5.2 | 2.75 | 9.22 | 4.82 | 7.23 | 3.26 | 3.42 | 15.9 |
| HISTO_Entropy_log2 | 5.67 | 5.4 | 5.45 | 5.02 | 5.48 | 4.82 | 5.15 | 4.73 | 5.15 | 5.26 | 4.26 |
| GLCM_Homogeneity | 0.212 | 0.217 | 0.15 | 0.169 | 0.154 | 0.272 | 0.219 | 0.256 | 0.279 | 0.223 | 0.307 |
| GLCM_Energy | 0.00183 | 0.0017 | 0.0119 | 0.011 | 0.00976 | 0.00524 | 0.00441 | 0.00416 | 0.00449 | 0.0017 | 0.00675 |
| GLCM_Contrast | 142 | 112 | 247 | 180 | 374 | 77.8 | 213 | 125 | 51.5 | 114 | 68.5 |
| GLCM_Correlation | 0.581 | 0.516 | 0.228 | 0.121 | 0.207 | 0.317 | 0.207 | 0.285 | 0.637 | 0.435 | 0.276 |
| GLCM_Entropy_log2 | 9.32 | 9.58 | 6.6 | 6.69 | 6.91 | 8.01 | 8.25 | 8.43 | 8.09 | 9.63 | 7.8 |
| GLCM_Dissimilarity | 8.9 | 8.12 | 12.5 | 10.6 | 14.9 | 6.21 | 10.3 | 7.6 | 5.34 | 8 | 5.37 |
| GLRLM_SRE | 0.977 | 0.972 | 0.991 | 0.99 | 0.987 | 0.973 | 0.971 | 0.961 | 0.972 | 0.974 | 0.959 |
| GLRLM_LRE | 1.1 | 1.12 | 1.04 | 1.04 | 1.06 | 1.13 | 1.14 | 1.19 | 1.13 | 1.12 | 1.19 |
| GLRLM_LGRE | 0.00786 | 0.00214 | 0.0198 | 0.00774 | 0.0155 | 0.00205 | 0.00369 | 0.0039 | 0.0036 | 0.00158 | 0.00186 |
| GLRLM_HGRE | 1400 | 1240 | 1230 | 1940 | 1800 | 2470 | 2110 | 1630 | 1090 | 1530 | 2840 |
| GLRLM_SRLGE | 0.00778 | 0.0021 | 0.0198 | 0.00774 | 0.0146 | 0.00203 | 0.00367 | 0.00379 | 0.00354 | 0.00156 | 0.00184 |
| GLRLM_SRHGE | 1370 | 1200 | 1220 | 1920 | 1780 | 2400 | 2040 | 1560 | 1060 | 1490 | 2720 |
| GLRLM_LRLGE | 0.0082 | 0.00231 | 0.0199 | 0.00777 | 0.0191 | 0.00212 | 0.00377 | 0.00437 | 0.00386 | 0.00169 | 0.00192 |
| GLRLM_LRHGE | 1570 | 1390 | 1280 | 2030 | 1910 | 2820 | 2450 | 1950 | 1230 | 1730 | 3410 |
| GLRLM_GLNU | 17.9 | 43.7 | 3.89 | 5.63 | 5.23 | 41.3 | 19.3 | 41.6 | 23.3 | 79.3 | 47.9 |
| GLRLM_RLNU | 770 | 1440 | 142 | 153 | 199 | 865 | 469 | 750 | 636 | 2380 | 618 |
| GLRLM_RP | 0.969 | 0.963 | 0.988 | 0.986 | 0.982 | 0.963 | 0.96 | 0.949 | 0.963 | 0.965 | 0.945 |
| NGLDM_Coarseness | 0 | 0.00449 | 0.0409 | 0.0382 | 0.0309 | 0.00944 | 0.011 | 0.00592 | 0.0147 | 0.00295 | 0.00784 |
| NGLDM_Contrast | 0 | 0.362 | 0.859 | 0.55 | 0.789 | 0.143 | 0.432 | 0.207 | 0.178 | 0.261 | 0.132 |
| NGLDM_Busyness | 0 | 0.133 | 0.0176 | 0.0304 | 0.0195 | 0.0352 | 0.0284 | 0.0625 | 0.041 | 0.128 | 0.0716 |
| GLZLM_SZE | 0.78 | 0.758 | 0.913 | 0.872 | 0.841 | 0.776 | 0.766 | 0.696 | 0.733 | 0.753 | 0.705 |
| GLZLM_LZE | 3.54 | 4.58 | 1.63 | 1.65 | 2.01 | 5.14 | 7.84 | 10.3 | 4.47 | 4.2 | 14.5 |
| GLZLM_LGZE | 0.00956 | 0.00255 | 0.0222 | 0.00903 | 0.00827 | 0.00276 | 0.00552 | 0.00443 | 0.00443 | 0.00194 | 0.00331 |

| Patient | 45 | 46 | 47 | 48 | 49 | 50 | 51 | 52 | 53 | 54 | 55 |
| --- | --- | --- | --- | --- | --- | --- | --- | --- | --- | --- | --- |
| Lesion (benign=0,malignant=1) | 0 | 0 | 0 | 0 | 0 | 0 | 0 | 0 | 0 | 0 | 0 |
| minValue | 139 | 56 | 31 | 23 | 109 | 61 | 127 | 130 | 78 | 58 | 44 |
| meanValue | 230 | 130 | 102 | 150 | 186 | 146 | 299 | 298 | 162 | 93.7 | 107 |
| stdValue | 23.3 | 25.6 | 19.3 | 28.1 | 20.8 | 19.7 | 54.8 | 32.1 | 18.5 | 6.62 | 16.4 |
| maxValue | 272 | 179 | 141 | 205 | 222 | 193 | 405 | 353 | 201 | 113 | 142 |
| HISTO_Skewness | -1.33 | -0.532 | -0.676 | -0.818 | -1.18 | -0.951 | -0.803 | -1.69 | -0.818 | -0.406 | -0.808 |
| HISTO_Kurtosis | 5 | 2.83 | 3.36 | 3.47 | 4.34 | 3.98 | 3.14 | 7.11 | 3.61 | 4.25 | 3.71 |
| HISTO_Entropy_log2 | 5.12 | 5.57 | 5.38 | 5.19 | 5.17 | 5.16 | 5.46 | 4.89 | 5.18 | 4.73 | 5.26 |
| GLCM_Homogeneity | 0.241 | 0.154 | 0.18 | 0.225 | 0.168 | 0.242 | 0.171 | 0.219 | 0.22 | 0.252 | 0.219 |
| GLCM_Energy | 0.00491 | 0.00544 | 0.00158 | 0.00192 | 0.0111 | 0.00168 | 0.00292 | 0.00324 | 0.00209 | 0.00357 | 0.00192 |
| GLCM_Contrast | 101 | 280 | 214 | 105 | 191 | 96.2 | 290 | 159 | 148 | 62.8 | 142 |
| GLCM_Correlation | 0.424 | 0.111 | 0.207 | 0.446 | 0.00537 | 0.464 | 0.201 | 0.195 | 0.242 | 0.331 | 0.374 |
| GLCM_Entropy_log2 | 7.92 | 7.74 | 9.57 | 9.41 | 6.91 | 9.81 | 8.59 | 8.56 | 9.26 | 8.48 | 9.57 |
| GLCM_Dissimilarity | 7.17 | 13 | 11.1 | 7.8 | 10.8 | 7.14 | 12.5 | 8.8 | 8.76 | 6.05 | 8.63 |
| GLRLM_SRE | 0.977 | 0.987 | 0.981 | 0.973 | 0.987 | 0.966 | 0.984 | 0.976 | 0.975 | 0.968 | 0.971 |
| GLRLM_LRE | 1.11 | 1.05 | 1.08 | 1.13 | 1.06 | 1.15 | 1.07 | 1.1 | 1.11 | 1.14 | 1.13 |
| GLRLM_LGRE | 0.00698 | 0.0052 | 0.00203 | 0.00129 | 0.00437 | 0.00167 | 0.00431 | 0.0019 | 0.00127 | 0.00147 | 0.00213 |
| GLRLM_HGRE | 2080 | 1700 | 1850 | 2120 | 2080 | 1830 | 1760 | 2460 | 2020 | 1830 | 1830 |
| GLRLM_SRLGE | 0.0068 | 0.00518 | 0.00201 | 0.00128 | 0.00436 | 0.00158 | 0.0043 | 0.00189 | 0.00126 | 0.00145 | 0.00211 |
| GLRLM_SRHGE | 2030 | 1680 | 1810 | 2050 | 2050 | 1770 | 1720 | 2400 | 1960 | 1770 | 1770 |
| GLRLM_LRLGE | 0.0077 | 0.00525 | 0.00208 | 0.00136 | 0.00442 | 0.00209 | 0.00436 | 0.00194 | 0.00133 | 0.00155 | 0.00223 |
| GLRLM_LRHGE | 2310 | 1790 | 2010 | 2410 | 2200 | 2130 | 1900 | 2730 | 2260 | 2090 | 2090 |
| GLRLM_GLNU | 12.6 | 9.48 | 37.6 | 62.3 | 10.7 | 221 | 13 | 33 | 60.6 | 55.4 | 66.1 |
| GLRLM_RLNU | 333 | 377 | 1250 | 1820 | 299 | 5970 | 450 | 739 | 1750 | 1150 | 1860 |
| GLRLM_RP | 0.968 | 0.984 | 0.974 | 0.963 | 0.983 | 0.955 | 0.978 | 0.969 | 0.967 | 0.958 | 0.962 |
| NGLDM_Coarseness | 0.0214 | 0.0236 | 0.00446 | 0.00376 | 0.0201 | 0.00109 | 0.0113 | 0.0077 | 0.00414 | 0.00528 | 0.00335 |
| NGLDM_Contrast | 0.378 | 0.475 | 0.453 | 0.293 | 0.49 | 0.218 | 0.677 | 0.269 | 0.245 | 0.161 | 0.315 |
| NGLDM_Busyness | 0.0205 | 0.0178 | 0.0782 | 0.112 | 0.0256 | 0.285 | 0.0342 | 0.0501 | 0.0864 | 0.131 | 0.0988 |
| GLZLM_SZE | 0.784 | 0.851 | 0.801 | 0.748 | 0.866 | 0.704 | 0.828 | 0.772 | 0.777 | 0.725 | 0.768 |
| GLZLM_LZE | 4.12 | 1.87 | 2.75 | 4.66 | 1.97 | 7.14 | 2.45 | 3.2 | 4.19 | 7.44 | 4.99 |
| GLZLM_LGZE | 0.00621 | 0.00613 | 0.00248 | 0.00173 | 0.00519 | 0.0015 | 0.00543 | 0.00255 | 0.00162 | 0.00211 | 0.00286 |

| Patient | 56 | 57 | 58 | 59 | 60 | 61 | 62 | 63 | 64 | 65 | 66 |
| --- | --- | --- | --- | --- | --- | --- | --- | --- | --- | --- | --- |
| Lesion (benign=0,malignant=1) | 0 | 0 | 0 | 0 | 0 | 0 | 0 | 0 | 0 | 0 | 0 |
| minValue | 114 | 77 | 413 | 122 | 162 | 70 | 125 | 85 | 66.6 | 109 | 54.6 |
| meanValue | 220 | 128 | 555 | 221 | 369 | 105 | 146 | 147 | 210 | 415 | 92.6 |
| stdValue | 16.5 | 11.3 | 44.2 | 49.3 | 75.5 | 11.4 | 6.85 | 15.7 | 28.2 | 72.3 | 7.14 |
| maxValue | 258 | 152 | 631 | 347 | 538 | 142 | 165 | 195 | 249 | 594 | 108 |
| HISTO_Skewness | -1.79 | -1.08 | -0.689 | 0.126 | -0.476 | -0.224 | -0.414 | 0.0399 | -2.56 | -1.05 | -1.61 |
| HISTO_Kurtosis | 9.4 | 4.94 | 3.29 | 2.04 | 2.59 | 3.41 | 3.73 | 3.28 | 10.9 | 3.97 | 6.48 |
| HISTO_Entropy_log2 | 4.6 | 5 | 5.05 | 5.6 | 5.53 | 5.21 | 4.64 | 5.09 | 4.67 | 5.11 | 4.84 |
| GLCM_Homogeneity | 0.278 | 0.207 | 0.148 | 0.137 | 0.21 | 0.201 | 0.233 | 0.314 | 0.304 | 0.264 | 0.324 |
| GLCM_Energy | 0.00453 | 0.00339 | 0.0146 | 0.0182 | 0.00296 | 0.0051 | 0.0106 | 0.00328 | 0.00651 | 0.00216 | 0.00454 |
| GLCM_Contrast | 71.1 | 193 | 412 | 211 | 96.3 | 127 | 101 | 46 | 105 | 105 | 60.6 |
| GLCM_Correlation | 0.304 | 0.122 | -0.212 | 0.196 | 0.491 | 0.397 | 0.313 | 0.682 | 0.481 | 0.408 | 0.546 |
| GLCM_Entropy_log2 | 8.29 | 8.51 | 6.23 | 6.31 | 8.6 | 7.9 | 6.86 | 8.7 | 7.69 | 9.65 | 8.41 |
| GLCM_Dissimilarity | 5.81 | 9.98 | 15.6 | 12 | 7.77 | 8.7 | 7.8 | 4.75 | 6.28 | 7.2 | 4.87 |
| GLRLM_SRE | 0.962 | 0.977 | 0.987 | 0.99 | 0.981 | 0.984 | 0.973 | 0.951 | 0.958 | 0.96 | 0.951 |
| GLRLM_LRE | 1.17 | 1.1 | 1.05 | 1.04 | 1.08 | 1.07 | 1.12 | 1.23 | 1.2 | 1.2 | 1.23 |
| GLRLM_LGRE | 0.00169 | 0.00259 | 0.0122 | 0.00876 | 0.00626 | 0.00399 | 0.00651 | 0.00182 | 0.00295 | 0.00119 | 0.00158 |
| GLRLM_HGRE | 2310 | 2030 | 1950 | 1020 | 1440 | 1090 | 1310 | 1420 | 2690 | 1750 | 2210 |
| GLRLM_SRLGE | 0.00168 | 0.00257 | 0.0122 | 0.00871 | 0.00611 | 0.00397 | 0.00647 | 0.00179 | 0.00293 | 0.00115 | 0.00156 |
| GLRLM_SRHGE | 2210 | 1980 | 1920 | 1010 | 1420 | 1070 | 1270 | 1340 | 2570 | 1670 | 2090 |
| GLRLM_LRLGE | 0.00177 | 0.00264 | 0.0122 | 0.00897 | 0.00687 | 0.00409 | 0.00669 | 0.00199 | 0.00306 | 0.00134 | 0.00168 |
| GLRLM_LRHGE | 2720 | 2240 | 2060 | 1060 | 1560 | 1170 | 1460 | 1780 | 3280 | 2150 | 2760 |
| GLRLM_GLNU | 50.3 | 23.6 | 2.98 | 5.34 | 14.5 | 17.7 | 11.7 | 42.6 | 27.5 | 251 | 63.6 |
| GLRLM_RLNU | 853 | 553 | 85.5 | 226 | 561 | 497 | 223 | 1050 | 475 | 6360 | 1220 |
| GLRLM_RP | 0.95 | 0.97 | 0.983 | 0.987 | 0.975 | 0.979 | 0.963 | 0.935 | 0.945 | 0.946 | 0.935 |
| NGLDM_Coarseness | 0.00628 | 0.00898 | 0.0366 | 0.0402 | 0.0163 | 0.0145 | 0.0176 | 0.0088 | 0.0138 | 0.00108 | 0.00577 |
| NGLDM_Contrast | 0.151 | 0.375 | 1.25 | 0.614 | 0.397 | 0.31 | 0.382 | 0.155 | 0.251 | 0.193 | 0.139 |
| NGLDM_Busyness | 0.0657 | 0.054 | 0.0372 | 0.0207 | 0.0299 | 0.0406 | 0.0739 | 0.0597 | 0.0187 | 0.269 | 0.0462 |
| GLZLM_SZE | 0.708 | 0.798 | 0.846 | 0.854 | 0.808 | 0.817 | 0.734 | 0.68 | 0.718 | 0.704 | 0.677 |
| GLZLM_LZE | 10.7 | 3.39 | 1.86 | 1.68 | 2.72 | 2.23 | 4.16 | 15 | 10.8 | 12 | 16.3 |
| GLZLM_LGZE | 0.00282 | 0.00341 | 0.0148 | 0.00951 | 0.00539 | 0.00473 | 0.0093 | 0.00296 | 0.00506 | 0.00148 | 0.00285 |

| Patient | 67 | 68 | 69 | 70 | 71 | 72 | 73 | 74 | 75 | 76 | 77 |
| --- | --- | --- | --- | --- | --- | --- | --- | --- | --- | --- | --- |
| Lesion (benign=0,malignant=1) | 0 | 0 | 0 | 0 | 0 | 1 | 1 | 1 | 1 | 1 | 1 |
| minValue | 137 | 261 | 232 | 88 | 143 | 6 | 186 | 127 | 108 | 93 | 139 |
| meanValue | 239 | 645 | 334 | 213 | 338 | 99.3 | 358 | 432 | 251 | 439 | 353 |
| stdValue | 27 | 43.6 | 19.8 | 27.3 | 36.5 | 19.2 | 53.4 | 59 | 30 | 71.1 | 48 |
| maxValue | 322 | 760 | 381 | 277 | 420 | 156 | 518 | 617 | 339 | 548 | 440 |
| HISTO_Skewness | -0.203 | -1.66 | -0.937 | -0.968 | -1.09 | 0.0368 | 0.0568 | -0.643 | -0.588 | -1.45 | -1.26 |
| HISTO_Kurtosis | 3.86 | 9.91 | 4.43 | 4.29 | 4.74 | 3.98 | 3.5 | 4.68 | 5.64 | 5.21 | 5.21 |
| HISTO_Entropy_log2 | 5.17 | 4.33 | 5.03 | 5.12 | 4.98 | 4.97 | 5.32 | 4.91 | 4.74 | 4.95 | 5.11 |
| GLCM_Homogeneity | 0.241 | 0.371 | 0.284 | 0.236 | 0.272 | 0.258 | 0.194 | 0.276 | 0.304 | 0.297 | 0.224 |
| GLCM_Energy | 0.00371 | 0.00716 | 0.00247 | 0.00171 | 0.00222 | 0.00274 | 0.00266 | 0.00243 | 0.00616 | 0.00489 | 0.00333 |
| GLCM_Contrast | 120 | 26.7 | 59 | 94.7 | 66.4 | 75.9 | 140 | 62.9 | 47.1 | 67.4 | 117 |
| GLCM_Correlation | 0.286 | 0.444 | 0.531 | 0.484 | 0.53 | 0.378 | 0.258 | 0.391 | 0.507 | 0.446 | 0.346 |
| GLCM_Entropy_log2 | 8.32 | 7.8 | 9.21 | 9.73 | 9.51 | 8.92 | 8.77 | 9.19 | 7.88 | 8.22 | 8.49 |
| GLCM_Dissimilarity | 7.97 | 3.6 | 5.55 | 7.25 | 5.88 | 6.36 | 9.07 | 5.78 | 4.98 | 5.62 | 8.06 |
| GLRLM_SRE | 0.974 | 0.936 | 0.957 | 0.969 | 0.956 | 0.964 | 0.982 | 0.955 | 0.956 | 0.958 | 0.975 |
| GLRLM_LRE | 1.14 | 1.36 | 1.2 | 1.13 | 1.2 | 1.16 | 1.08 | 1.21 | 1.22 | 1.2 | 1.11 |
| GLRLM_LGRE | 0.00273 | 0.000949 | 0.000996 | 0.00103 | 0.00082 | 0.00169 | 0.00516 | 0.00143 | 0.000759 | 0.00212 | 0.00252 |
| GLRLM_HGRE | 1370 | 2490 | 2020 | 1930 | 2130 | 1700 | 1230 | 1690 | 1820 | 2500 | 2210 |
| GLRLM_SRLGE | 0.00269 | 0.000924 | 0.000973 | 0.00101 | 0.000799 | 0.00167 | 0.00514 | 0.0014 | 0.000734 | 0.00211 | 0.00251 |
| GLRLM_SRHGE | 1330 | 2320 | 1930 | 1870 | 2030 | 1640 | 1210 | 1610 | 1740 | 2370 | 2150 |
| GLRLM_LRLGE | 0.00289 | 0.00109 | 0.0011 | 0.00111 | 0.000916 | 0.0018 | 0.00524 | 0.00158 | 0.000885 | 0.00219 | 0.00257 |
| GLRLM_LRHGE | 1560 | 3440 | 2460 | 2210 | 2600 | 1950 | 1330 | 2050 | 2210 | 3070 | 2500 |
| GLRLM_GLNU | 25.9 | 121 | 128 | 143 | 432 | 40.7 | 16.7 | 135 | 21.6 | 26.6 | 20.4 |
| GLRLM_RLNU | 730 | 1710 | 3080 | 3760 | 9910 | 957 | 536 | 3080 | 399 | 584 | 539 |
| GLRLM_RP | 0.964 | 0.914 | 0.943 | 0.96 | 0.941 | 0.952 | 0.976 | 0.939 | 0.94 | 0.943 | 0.966 |
| NGLDM_Coarseness | 0.0127 | 0.00426 | 0.00265 | 0.00182 | 0.000758 | 0.00655 | 0.0119 | 0.0021 | 0.0166 | 0.0125 | 0.0121 |
| NGLDM_Contrast | 0.211 | 0.0772 | 0.147 | 0.208 | 0.131 | 0.238 | 0.36 | 0.142 | 0.166 | 0.297 | 0.33 |
| NGLDM_Busyness | 0.0378 | 0.0816 | 0.119 | 0.169 | 0.347 | 0.113 | 0.0452 | 0.177 | 0.0399 | 0.0347 | 0.0329 |
| GLZLM_SZE | 0.738 | 0.649 | 0.679 | 0.725 | 0.685 | 0.721 | 0.786 | 0.689 | 0.733 | 0.794 | 0.777 |
| GLZLM_LZE | 4.49 | 40.5 | 10.2 | 5.76 | 16.7 | 8.01 | 2.64 | 20.2 | 8.55 | 12.7 | 4.17 |
| GLZLM_LGZE | 0.00352 | 0.00181 | 0.00144 | 0.00128 | 0.00116 | 0.00248 | 0.00666 | 0.0022 | 0.000908 | 0.00352 | 0.00358 |

| Patient | 78 | 79 | 80 | 81 | 82 | 83 | 84 | 85 | 86 | 87 | 88 |
| --- | --- | --- | --- | --- | --- | --- | --- | --- | --- | --- | --- |
| Lesion (benign=0,malignant=1) | 1 | 1 | 1 | 1 | 1 | 1 | 1 | 1 | 1 | 1 | 1 |
| minValue | 29 | 77 | 33 | 70 | 37 | 49 | 127 | 7 | 30 | 133 | 103 |
| meanValue | 326 | 457 | 195 | 463 | 510 | 299 | 539 | 75.3 | 210 | 481 | 343 |
| stdValue | 70 | 61.8 | 44.6 | 116 | 108 | 87.3 | 116 | 13.9 | 59.5 | 82.7 | 45.1 |
| maxValue | 470 | 585 | 291 | 606 | 705 | 481 | 742 | 102 | 313 | 623 | 434 |
| HISTO_Skewness | -1.24 | -1.5 | -0.515 | -1.38 | -1.49 | -0.491 | -1.05 | -1.55 | -0.586 | -2.14 | -1.63 |
| HISTO_Kurtosis | 4.73 | 7.31 | 3.18 | 4.26 | 5.36 | 2.75 | 3.28 | 6.24 | 2.64 | 8.18 | 6.96 |
| HISTO_Entropy_log2 | 5.17 | 4.79 | 5.43 | 5.21 | 5.09 | 5.63 | 5.27 | 4.92 | 5.62 | 4.85 | 4.89 |
| GLCM_Homogeneity | 0.239 | 0.29 | 0.234 | 0.263 | 0.256 | 0.187 | 0.256 | 0.266 | 0.209 | 0.264 | 0.286 |
| GLCM_Energy | 0.00203 | 0.0035 | 0.00199 | 0.00494 | 0.00269 | 0.00161 | 0.00291 | 0.00306 | 0.00133 | 0.00638 | 0.00354 |
| GLCM_Contrast | 95.2 | 54 | 136 | 132 | 114 | 167 | 136 | 74.9 | 175 | 97.1 | 64.1 |
| GLCM_Correlation | 0.389 | 0.419 | 0.317 | 0.434 | 0.288 | 0.478 | 0.388 | 0.38 | 0.382 | 0.269 | 0.349 |
| GLCM_Entropy_log2 | 9.47 | 8.55 | 9.32 | 8.14 | 9.26 | 9.49 | 9.13 | 8.99 | 9.96 | 7.68 | 8.7 |
| GLCM_Dissimilarity | 7.17 | 5.22 | 8.38 | 7.74 | 7.33 | 9.94 | 7.95 | 6.16 | 9.72 | 6.8 | 5.5 |
| GLRLM_SRE | 0.97 | 0.96 | 0.968 | 0.967 | 0.964 | 0.984 | 0.964 | 0.961 | 0.971 | 0.972 | 0.959 |
| GLRLM_LRE | 1.13 | 1.18 | 1.15 | 1.15 | 1.17 | 1.07 | 1.17 | 1.18 | 1.13 | 1.13 | 1.18 |
| GLRLM_LGRE | 0.00144 | 0.00145 | 0.00188 | 0.00602 | 0.00127 | 0.00439 | 0.00179 | 0.00152 | 0.00306 | 0.00852 | 0.0013 |
| GLRLM_HGRE | 1990 | 2390 | 1770 | 2410 | 2190 | 1580 | 2000 | 2240 | 1850 | 2220 | 2260 |
| GLRLM_SRLGE | 0.00142 | 0.00143 | 0.00186 | 0.00599 | 0.00125 | 0.00436 | 0.00177 | 0.00148 | 0.00301 | 0.00837 | 0.00128 |
| GLRLM_SRHGE | 1920 | 2290 | 1700 | 2310 | 2100 | 1550 | 1910 | 2140 | 1780 | 2150 | 2160 |
| GLRLM_LRLGE | 0.00157 | 0.00152 | 0.00197 | 0.00613 | 0.00136 | 0.00452 | 0.00187 | 0.0017 | 0.00328 | 0.00914 | 0.00137 |
| GLRLM_LRHGE | 2300 | 2870 | 2070 | 2870 | 2620 | 1700 | 2440 | 2670 | 2160 | 2550 | 2720 |
| GLRLM_GLNU | 112 | 47.7 | 33.4 | 18.8 | 128 | 27.7 | 46.3 | 136 | 46.3 | 21.3 | 79.1 |
| GLRLM_RLNU | 3040 | 986 | 1140 | 478 | 3100 | 1170 | 1300 | 2860 | 1900 | 410 | 1690 |
| GLRLM_RP | 0.96 | 0.946 | 0.957 | 0.956 | 0.951 | 0.979 | 0.95 | 0.948 | 0.962 | 0.962 | 0.946 |
| NGLDM_Coarseness | 0.00233 | 0.00726 | 0.00588 | 0.0126 | 0.00188 | 0.00641 | 0.00457 | 0.00207 | 0.00341 | 0.0156 | 0.00416 |
| NGLDM_Contrast | 0.236 | 0.164 | 0.375 | 0.536 | 0.278 | 0.516 | 0.434 | 0.18 | 0.62 | 0.278 | 0.161 |
| NGLDM_Busyness | 0.117 | 0.06 | 0.0658 | 0.0217 | 0.138 | 0.067 | 0.066 | 0.114 | 0.112 | 0.0187 | 0.0656 |
| GLZLM_SZE | 0.759 | 0.712 | 0.74 | 0.765 | 0.763 | 0.817 | 0.791 | 0.714 | 0.758 | 0.761 | 0.731 |
| GLZLM_LZE | 6.04 | 9.03 | 5.56 | 7.5 | 12.1 | 2.33 | 15.5 | 15.3 | 5.58 | 4.9 | 14.1 |
| GLZLM_LGZE | 0.00178 | 0.00233 | 0.00269 | 0.00921 | 0.00177 | 0.00517 | 0.00266 | 0.00178 | 0.00357 | 0.00976 | 0.00206 |

| Patient | 89 | 90 | 91 | 92 | 93 | 94 | 95 | 96 | 97 | 98 | 99 |
| --- | --- | --- | --- | --- | --- | --- | --- | --- | --- | --- | --- |
| Lesion (benign=0,malignant=1) | 1 | 1 | 1 | 1 | 1 | 1 | 1 | 1 | 1 | 1 | 1 |
| minValue | 56 | 0 | 56 | 76 | 299 | 107 | 61 | 92 | 71 | 218 | 26 |
| meanValue | 130 | 161 | 366 | 370 | 418 | 300 | 238 | 409 | 254 | 361 | 192 |
| stdValue | 20.1 | 31.7 | 95.2 | 113 | 27.7 | 83.4 | 33.3 | 84.2 | 32 | 45.2 | 37.9 |
| maxValue | 162 | 277 | 567 | 586 | 467 | 499 | 309 | 593 | 373 | 474 | 297 |
| HISTO_Skewness | -1.13 | -0.794 | -0.797 | -0.205 | -1.35 | -0.0521 | -1.08 | -0.913 | -0.712 | -0.36 | -0.352 |
| HISTO_Kurtosis | 4.33 | 6.56 | 3.27 | 2.28 | 6.05 | 2.44 | 4.79 | 3.77 | 5.1 | 2.88 | 3.93 |
| HISTO_Entropy_log2 | 5.26 | 4.76 | 5.44 | 5.6 | 4.46 | 5.5 | 5 | 5.23 | 4.69 | 5.43 | 5.14 |
| GLCM_Homogeneity | 0.221 | 0.359 | 0.205 | 0.16 | 0.29 | 0.149 | 0.262 | 0.21 | 0.291 | 0.178 | 0.238 |
| GLCM_Energy | 0.00421 | 0.00444 | 0.00159 | 0.00405 | 0.00985 | 0.00647 | 0.00279 | 0.00288 | 0.00404 | 0.00259 | 0.00245 |
| GLCM_Contrast | 124 | 31.4 | 176 | 267 | 50.6 | 269 | 90 | 189 | 51.3 | 187 | 100 |
| GLCM_Correlation | 0.354 | 0.625 | 0.276 | 0.283 | 0.431 | 0.328 | 0.248 | 0.122 | 0.293 | 0.167 | 0.25 |
| GLCM_Entropy_log2 | 8.11 | 8.64 | 9.61 | 8.05 | 6.81 | 7.4 | 9.08 | 8.8 | 8.61 | 8.77 | 9.11 |
| GLCM_Dissimilarity | 8.26 | 3.75 | 9.64 | 12.8 | 5.17 | 13.1 | 6.75 | 9.93 | 5.18 | 10.8 | 7.28 |
| GLRLM_SRE | 0.976 | 0.929 | 0.977 | 0.983 | 0.947 | 0.988 | 0.962 | 0.976 | 0.955 | 0.981 | 0.971 |
| GLRLM_LRE | 1.1 | 1.35 | 1.1 | 1.07 | 1.21 | 1.05 | 1.18 | 1.11 | 1.21 | 1.08 | 1.13 |
| GLRLM_LGRE | 0.00443 | 0.00211 | 0.00339 | 0.00923 | 0.000433 | 0.0101 | 0.00106 | 0.00274 | 0.0012 | 0.00517 | 0.00146 |
| GLRLM_HGRE | 2170 | 1470 | 1610 | 1590 | 2660 | 1210 | 2200 | 1790 | 1580 | 1460 | 1650 |
| GLRLM_SRLGE | 0.00442 | 0.002 | 0.00333 | 0.00898 | 0.000414 | 0.01 | 0.00105 | 0.00273 | 0.00117 | 0.00505 | 0.00144 |
| GLRLM_SRHGE | 2100 | 1360 | 1560 | 1560 | 2510 | 1190 | 2110 | 1740 | 1510 | 1430 | 1600 |
| GLRLM_LRLGE | 0.00447 | 0.0026 | 0.00367 | 0.0102 | 0.00051 | 0.0101 | 0.00114 | 0.00282 | 0.00134 | 0.00569 | 0.00156 |
| GLRLM_LRHGE | 2430 | 1990 | 1800 | 1750 | 3270 | 1280 | 2640 | 2010 | 1920 | 1580 | 1880 |
| GLRLM_GLNU | 11.7 | 548 | 37.7 | 5.87 | 4.58 | 4.97 | 80.4 | 25.9 | 112 | 13.8 | 59 |
| GLRLM_RLNU | 344 | 9590 | 1320 | 244 | 77.1 | 189 | 1940 | 717 | 2020 | 492 | 1590 |
| GLRLM_RP | 0.968 | 0.906 | 0.969 | 0.977 | 0.934 | 0.985 | 0.949 | 0.967 | 0.94 | 0.975 | 0.961 |
| NGLDM_Coarseness | 0.0182 | 0.000838 | 0.00488 | 0.0213 | 0.0643 | 0.0292 | 0.00329 | 0.00772 | 0.00317 | 0.0108 | 0.00442 |
| NGLDM_Contrast | 0.451 | 0.066 | 0.478 | 0.988 | 0.414 | 0.823 | 0.215 | 0.439 | 0.122 | 0.563 | 0.216 |
| NGLDM_Busyness | 0.0223 | 0.345 | 0.0817 | 0.028 | 0.0364 | 0.0265 | 0.111 | 0.0512 | 0.148 | 0.0482 | 0.0943 |
| GLZLM_SZE | 0.806 | 0.673 | 0.791 | 0.813 | 0.768 | 0.895 | 0.76 | 0.789 | 0.7 | 0.8 | 0.718 |
| GLZLM_LZE | 3.67 | 183 | 3.59 | 2.36 | 2.21 | 1.76 | 16.5 | 4.65 | 17.2 | 2.69 | 4.6 |
| GLZLM_LGZE | 0.00607 | 0.00254 | 0.0035 | 0.00696 | 0.000453 | 0.0116 | 0.00152 | 0.00373 | 0.00176 | 0.00425 | 0.00192 |

| Patient | 100 | 101 | 102 | 103 | 104 | 105 | 106 | 107 | 108 | 109 | 110 |
| --- | --- | --- | --- | --- | --- | --- | --- | --- | --- | --- | --- |
| Lesion (benign=0,malignant=1) | 1 | 1 | 1 | 1 | 1 | 1 | 1 | 1 | 1 | 1 | 1 |
| minValue | 23 | 33 | 6 | 90 | 58 | 36 | 8 | 20 | 79 | 88 | 39 |
| meanValue | 139 | 154 | 118 | 295 | 474 | 146 | 80 | 179 | 206 | 124 | 147 |
| stdValue | 41 | 41.6 | 21.7 | 52.1 | 78.2 | 26 | 20.8 | 44.7 | 41.1 | 13 | 41.8 |
| maxValue | 234 | 244 | 163 | 366 | 596 | 203 | 131 | 259 | 297 | 163 | 238 |
| HISTO_Skewness | -0.166 | -0.468 | -1.91 | -1.32 | -1.59 | -1.55 | -0.621 | -0.883 | -0.536 | 0.428 | -0.041 |
| HISTO_Kurtosis | 2.3 | 2.49 | 7.77 | 4.86 | 6.11 | 6.11 | 2.99 | 3.03 | 2.59 | 4 | 2.21 |
| HISTO_Entropy_log2 | 5.46 | 5.55 | 4.79 | 5.19 | 4.89 | 5 | 5.38 | 5.35 | 5.47 | 4.85 | 5.67 |
| GLCM_Homogeneity | 0.169 | 0.22 | 0.288 | 0.242 | 0.264 | 0.262 | 0.238 | 0.27 | 0.202 | 0.185 | 0.169 |
| GLCM_Energy | 0.00383 | 0.00225 | 0.00372 | 0.00373 | 0.00402 | 0.00357 | 0.00162 | 0.00282 | 0.00174 | 0.0147 | 0.00153 |
| GLCM_Contrast | 255 | 138 | 65.8 | 104 | 94.1 | 109 | 95 | 79.8 | 169 | 186 | 216 |
| GLCM_Correlation | 0.0323 | 0.515 | 0.378 | 0.538 | 0.244 | 0.188 | 0.489 | 0.593 | 0.342 | 0.187 | 0.364 |
| GLCM_Entropy_log2 | 8.2 | 9.26 | 8.86 | 8.38 | 8.52 | 8.7 | 9.72 | 9.13 | 9.48 | 6.23 | 9.53 |
| GLCM_Dissimilarity | 12.5 | 8.71 | 5.57 | 7.44 | 6.73 | 6.99 | 7.23 | 6.37 | 9.81 | 10.5 | 11.4 |
| GLRLM_SRE | 0.98 | 0.977 | 0.954 | 0.97 | 0.964 | 0.965 | 0.969 | 0.963 | 0.976 | 0.984 | 0.983 |
| GLRLM_LRE | 1.09 | 1.1 | 1.21 | 1.13 | 1.17 | 1.17 | 1.14 | 1.17 | 1.11 | 1.07 | 1.07 |
| GLRLM_LGRE | 0.00501 | 0.00281 | 0.00121 | 0.00304 | 0.00152 | 0.00275 | 0.00166 | 0.0019 | 0.00269 | 0.0123 | 0.00405 |
| GLRLM_HGRE | 1420 | 1530 | 2210 | 2420 | 2570 | 1910 | 1550 | 1970 | 1560 | 1090 | 1430 |
| GLRLM_SRLGE | 0.00499 | 0.00278 | 0.00118 | 0.00302 | 0.00151 | 0.00271 | 0.00164 | 0.00188 | 0.00267 | 0.0122 | 0.00396 |
| GLRLM_SRHGE | 1390 | 1490 | 2100 | 2330 | 2460 | 1830 | 1490 | 1880 | 1520 | 1070 | 1400 |
| GLRLM_LRLGE | 0.00509 | 0.00294 | 0.00132 | 0.0031 | 0.00158 | 0.0029 | 0.00177 | 0.00198 | 0.00278 | 0.0123 | 0.00443 |
| GLRLM_LRHGE | 1560 | 1740 | 2720 | 2830 | 3050 | 2250 | 1800 | 2380 | 1770 | 1160 | 1550 |
| GLRLM_GLNU | 9.84 | 30.3 | 348 | 16.5 | 47.5 | 48 | 77.9 | 50.6 | 28.9 | 4.26 | 18.6 |
| GLRLM_RLNU | 363 | 1180 | 6490 | 479 | 1010 | 1050 | 2600 | 1570 | 1050 | 92.1 | 814 |
| GLRLM_RP | 0.974 | 0.969 | 0.939 | 0.96 | 0.951 | 0.952 | 0.959 | 0.951 | 0.968 | 0.979 | 0.977 |
| NGLDM_Coarseness | 0.0138 | 0.00747 | 0.000938 | 0.0135 | 0.00588 | 0.00599 | 0.00294 | 0.00487 | 0.00603 | 0.0408 | 0.00746 |
| NGLDM_Contrast | 0.773 | 0.396 | 0.147 | 0.487 | 0.279 | 0.265 | 0.294 | 0.344 | 0.491 | 0.725 | 0.696 |
| NGLDM_Busyness | 0.0481 | 0.0548 | 0.242 | 0.0339 | 0.0581 | 0.0501 | 0.129 | 0.0645 | 0.0675 | 0.0347 | 0.0767 |
| GLZLM_SZE | 0.809 | 0.817 | 0.718 | 0.775 | 0.77 | 0.738 | 0.76 | 0.778 | 0.784 | 0.822 | 0.822 |
| GLZLM_LZE | 2.86 | 4.82 | 23.9 | 5.68 | 8.57 | 9.54 | 5.53 | 11.5 | 3.82 | 2.16 | 2.58 |
| GLZLM_LGZE | 0.00646 | 0.00328 | 0.0018 | 0.00453 | 0.00235 | 0.00406 | 0.00216 | 0.00282 | 0.00353 | 0.0154 | 0.00171 |

| Patient | 111 | 112 | 113 | 114 | 115 | 116 | 117 | 118 | 119 | 120 | 121 |
| --- | --- | --- | --- | --- | --- | --- | --- | --- | --- | --- | --- |
| Lesion (benign=0,malignant=1) | 1 | 1 | 1 | 1 | 1 | 1 | 1 | 1 | 1 | 1 | 1 |
| minValue | 23 | 90 | 58 | 93 | 14 | 3 | 38 | 23 | 32 | 12 | 39 |
| meanValue | 94.2 | 292 | 107 | 436 | 105 | 30.4 | 282 | 111 | 247 | 95.3 | 208 |
| stdValue | 16.1 | 53.6 | 15.2 | 101 | 25.8 | 8.18 | 54.8 | 20.6 | 73.5 | 18.5 | 57.4 |
| maxValue | 131 | 411 | 137 | 610 | 168 | 59 | 393 | 156 | 416 | 138 | 361 |
| HISTO_Skewness | -0.696 | -0.586 | -0.449 | -0.923 | -0.547 | -0.231 | -1.17 | -0.897 | -0.435 | -1.5 | -0.563 |
| HISTO_Kurtosis | 3.71 | 3.33 | 3.05 | 3.21 | 2.77 | 2.72 | 4.46 | 3.79 | 2.62 | 6.46 | 2.85 |
| HISTO_Entropy_log2 | 5.17 | 5.37 | 5.09 | 5.46 | 5.35 | 5.06 | 5.11 | 5.21 | 5.57 | 4.95 | 5.46 |
| GLCM_Homogeneity | 0.22 | 0.226 | 0.188 | 0.221 | 0.244 | 0.282 | 0.258 | 0.259 | 0.196 | 0.286 | 0.219 |
| GLCM_Energy | 0.00188 | 0.00194 | 0.022 | 0.00169 | 0.00142 | 0.00205 | 0.0029 | 0.00222 | 0.00149 | 0.0036 | 0.00155 |
| GLCM_Contrast | 99.1 | 101 | 154 | 161 | 92.4 | 60.9 | 85.8 | 93.1 | 137 | 68.4 | 145 |
| GLCM_Correlation | 0.412 | 0.418 | 0.164 | 0.387 | 0.535 | 0.615 | 0.363 | 0.361 | 0.462 | 0.373 | 0.294 |
| GLCM_Entropy_log2 | 9.47 | 9.32 | 5.79 | 9.76 | 9.92 | 9.41 | 9 | 9.37 | 9.64 | 8.82 | 9.72 |
| GLCM_Dissimilarity | 7.66 | 7.56 | 9.92 | 9.08 | 7.04 | 5.67 | 6.72 | 6.83 | 9.06 | 5.71 | 8.92 |
| GLRLM_SRE | 0.972 | 0.972 | 0.987 | 0.969 | 0.963 | 0.95 | 0.96 | 0.963 | 0.978 | 0.955 | 0.971 |
| GLRLM_LRE | 1.12 | 1.12 | 1.06 | 1.14 | 1.17 | 1.23 | 1.19 | 1.17 | 1.09 | 1.21 | 1.13 |
| GLRLM_LGRE | 0.00131 | 0.0019 | 0.0102 | 0.00199 | 0.00177 | 0.00177 | 0.00143 | 0.00169 | 0.00331 | 0.00148 | 0.00447 |
| GLRLM_HGRE | 1910 | 1780 | 1770 | 1980 | 1570 | 1100 | 2070 | 1920 | 1480 | 1920 | 1280 |
| GLRLM_SRLGE | 0.0013 | 0.00188 | 0.0102 | 0.00197 | 0.00173 | 0.00171 | 0.00141 | 0.00165 | 0.00328 | 0.00145 | 0.0044 |
| GLRLM_SRHGE | 1850 | 1720 | 1740 | 1910 | 1510 | 1050 | 1970 | 1840 | 1440 | 1830 | 1240 |
| GLRLM_LRLGE | 0.00138 | 0.00197 | 0.0103 | 0.00208 | 0.00196 | 0.00207 | 0.00152 | 0.00187 | 0.00347 | 0.00161 | 0.00479 |
| GLRLM_LRHGE | 2160 | 2020 | 1880 | 2310 | 1870 | 1370 | 2490 | 2300 | 1630 | 2340 | 1480 |
| GLRLM_GLNU | 58.5 | 32.6 | 3.8 | 55.8 | 141 | 289 | 54.4 | 82.6 | 27.2 | 113 | 55.7 |
| GLRLM_RLNU | 1620 | 1090 | 104 | 1880 | 4560 | 7490 | 1370 | 2320 | 1080 | 2330 | 1980 |
| GLRLM_RP | 0.962 | 0.964 | 0.982 | 0.959 | 0.951 | 0.934 | 0.947 | 0.951 | 0.971 | 0.94 | 0.961 |
| NGLDM_Coarseness | 0.00418 | 0.0069 | 0.0507 | 0.00348 | 0.00161 | 0.000988 | 0.00465 | 0.00304 | 0.00732 | 0.003 | 0.00336 |
| NGLDM_Contrast | 0.258 | 0.299 | 0.676 | 0.48 | 0.276 | 0.157 | 0.27 | 0.245 | 0.424 | 0.171 | 0.394 |
| NGLDM_Busyness | 0.0939 | 0.054 | 0.0211 | 0.0875 | 0.221 | 0.537 | 0.0714 | 0.104 | 0.058 | 0.0867 | 0.127 |
| GLZLM_SZE | 0.742 | 0.76 | 0.83 | 0.764 | 0.723 | 0.686 | 0.719 | 0.743 | 0.784 | 0.72 | 0.747 |
| GLZLM_LZE | 5.15 | 4.39 | 1.97 | 7.06 | 8.74 | 26.1 | 11.1 | 9.73 | 3.39 | 16.8 | 4.92 |
| GLZLM_LGZE | 0.00174 | 0.0026 | 0.0126 | 0.00281 | 0.00217 | 0.00224 | 0.00215 | 0.00194 | 0.00392 | 0.0023 | 0.00566 |

| Patient | 122 | 123 | 124 | 125 | 126 | 127 | 128 | 129 | 130 | 131 | 132 |
| --- | --- | --- | --- | --- | --- | --- | --- | --- | --- | --- | --- |
| Lesion (benign=0,malignant=1) | 1 | 1 | 1 | 1 | 1 | 1 | 1 | 1 | 1 | 1 | 1 |
| minValue | 6 | 13 | 26 | 21 | 6 | 83 | 18 | 134 | 73 | 11 | 47 |
| meanValue | 73.6 | 132 | 242 | 113 | 117 | 425 | 157 | 308 | 319 | 53.1 | 318 |
| stdValue | 18.7 | 56.7 | 107 | 26.1 | 32.7 | 96.9 | 48.1 | 33.5 | 74.3 | 14.6 | 67.3 |
| maxValue | 118 | 271 | 467 | 262 | 178 | 664 | 252 | 475 | 511 | 81 | 433 |
| HISTO_Skewness | -1.04 | -0.0898 | 0.0808 | 0.382 | -0.971 | -0.662 | -0.269 | 0.76 | -0.652 | -0.825 | -1.29 |
| HISTO_Kurtosis | 3.94 | 1.82 | 2.15 | 5.67 | 3.44 | 3.02 | 2.43 | 7.87 | 3.54 | 2.89 | 4.27 |
| HISTO_Entropy_log2 | 5.22 | 5.65 | 5.84 | 4.74 | 5.42 | 5.33 | 5.62 | 4.39 | 5.36 | 5.48 | 5.16 |
| GLCM_Homogeneity | 0.255 | 0.225 | 0.165 | 0.318 | 0.246 | 0.24 | 0.191 | 0.371 | 0.232 | 0.194 | 0.274 |
| GLCM_Energy | 0.0023 | 0.00107 | 0.00125 | 0.00373 | 0.00173 | 0.0022 | 0.00163 | 0.00823 | 0.00224 | 0.00255 | 0.00338 |
| GLCM_Contrast | 86.5 | 158 | 360 | 39.6 | 122 | 115 | 257 | 26.1 | 108 | 154 | 92.1 |
| GLCM_Correlation | 0.47 | 0.617 | 0.239 | 0.57 | 0.479 | 0.324 | 0.257 | 0.489 | 0.334 | 0.351 | 0.395 |
| GLCM_Entropy_log2 | 9.42 | 10.4 | 9.88 | 8.91 | 9.8 | 9.24 | 9.5 | 7.68 | 9.29 | 8.91 | 8.75 |
| GLCM_Dissimilarity | 6.75 | 9.18 | 14.4 | 4.55 | 7.83 | 7.69 | 11.8 | 3.54 | 7.72 | 9.46 | 6.6 |
| GLRLM_SRE | 0.96 | 0.966 | 0.982 | 0.941 | 0.965 | 0.97 | 0.977 | 0.931 | 0.969 | 0.979 | 0.961 |
| GLRLM_LRE | 1.19 | 1.16 | 1.09 | 1.3 | 1.16 | 1.14 | 1.1 | 1.35 | 1.14 | 1.09 | 1.18 |
| GLRLM_LGRE | 0.00257 | 0.00417 | 0.0069 | 0.00324 | 0.00189 | 0.00215 | 0.00263 | 0.00191 | 0.00648 | 0.00621 | 0.00176 |
| GLRLM_HGRE | 1640 | 1110 | 1250 | 673 | 1860 | 1560 | 1640 | 1140 | 1450 | 1690 | 2170 |
| GLRLM_SRLGE | 0.00252 | 0.00401 | 0.00673 | 0.00308 | 0.00186 | 0.00213 | 0.0026 | 0.00184 | 0.00617 | 0.00611 | 0.00174 |
| GLRLM_SRHGE | 1560 | 1070 | 1230 | 633 | 1780 | 1510 | 1600 | 1070 | 1400 | 1650 | 2070 |
| GLRLM_LRLGE | 0.00282 | 0.00498 | 0.00778 | 0.004 | 0.00204 | 0.00225 | 0.00276 | 0.00227 | 0.00797 | 0.00663 | 0.00184 |
| GLRLM_LRHGE | 1990 | 1260 | 1370 | 870 | 2240 | 1810 | 1840 | 1510 | 1660 | 1880 | 2640 |
| GLRLM_GLNU | 113 | 87.4 | 34.6 | 618 | 97 | 36.6 | 21.5 | 82.3 | 40.2 | 18.4 | 38.1 |
| GLRLM_RLNU | 3070 | 3750 | 1780 | 11000 | 3140 | 1160 | 883 | 1090 | 1240 | 647 | 971 |
| GLRLM_RP | 0.946 | 0.954 | 0.975 | 0.921 | 0.953 | 0.959 | 0.969 | 0.907 | 0.959 | 0.972 | 0.947 |
| NGLDM_Coarseness | 0.00226 | 0.00181 | 0.00396 | 0.000591 | 0.00222 | 0.0062 | 0.00669 | 0.00578 | 0.00577 | 0.00989 | 0.00712 |
| NGLDM_Contrast | 0.265 | 0.613 | 0.869 | 0.075 | 0.395 | 0.324 | 0.682 | 0.0669 | 0.306 | 0.621 | 0.322 |
| NGLDM_Busyness | 0.142 | 0.318 | 0.168 | 0.759 | 0.144 | 0.061 | 0.0703 | 0.084 | 0.0658 | 0.0387 | 0.0431 |
| GLZLM_SZE | 0.717 | 0.768 | 0.815 | 0.641 | 0.775 | 0.782 | 0.777 | 0.702 | 0.766 | 0.801 | 0.784 |
| GLZLM_LZE | 13.3 | 13.2 | 2.97 | 51.6 | 10.3 | 6.05 | 3.62 | 104 | 6.02 | 3.21 | 11.5 |
| GLZLM_LGZE | 0.00338 | 0.00422 | 0.00651 | 0.00406 | 0.00241 | 0.003 | 0.0032 | 0.00354 | 0.00564 | 0.00655 | 0.00272 |

| Patient | 133 | 134 | 135 | 136 | 137 | 138 | 139 | 140 | 141 | 142 | 143 |
| --- | --- | --- | --- | --- | --- | --- | --- | --- | --- | --- | --- |
| Lesion (benign=0,malignant=1) | 1 | 1 | 1 | 1 | 1 | 1 | 1 | 1 | 1 | 1 | 1 |
| minValue | 62 | 11 | 18 | 54 | 5 | 54 | 63 | 14 | 41 | 84 | 31 |
| meanValue | 411 | 255 | 51 | 333 | 108 | 373 | 397 | 186 | 323 | 409 | 218 |
| stdValue | 118 | 64.4 | 7.18 | 66 | 23.1 | 116 | 101 | 57.1 | 62.4 | 103 | 47.6 |
| maxValue | 628 | 417 | 67 | 524 | 159 | 603 | 610 | 279 | 510 | 582 | 449 |
| HISTO_Skewness | -0.664 | -0.32 | -0.958 | -0.318 | -1.11 | -0.446 | -0.534 | -1.2 | -1.58 | -1.06 | 0.423 |
| HISTO_Kurtosis | 2.52 | 3.83 | 4.66 | 3.15 | 4.38 | 2.36 | 3.18 | 3.58 | 7.08 | 3.51 | 6.08 |
| HISTO_Entropy_log2 | 5.56 | 5.28 | 4.77 | 5.15 | 5.09 | 5.68 | 5.43 | 5.37 | 4.83 | 5.44 | 4.72 |
| GLCM_Homogeneity | 0.219 | 0.244 | 0.255 | 0.237 | 0.262 | 0.203 | 0.205 | 0.262 | 0.28 | 0.235 | 0.409 |
| GLCM_Energy | 0.00151 | 0.00263 | 0.00331 | 0.00216 | 0.00341 | 0.00116 | 0.00241 | 0.00252 | 0.00427 | 0.0025 | 0.00682 |
| GLCM_Contrast | 163 | 76.2 | 86.6 | 78.2 | 92 | 141 | 144 | 108 | 56.7 | 156 | 25.3 |
| GLCM_Correlation | 0.427 | 0.529 | 0.425 | 0.496 | 0.192 | 0.556 | 0.421 | 0.533 | 0.266 | 0.358 | 0.708 |
| GLCM_Entropy_log2 | 9.98 | 8.93 | 8.7 | 9.16 | 8.63 | 10.1 | 8.87 | 9.45 | 8.32 | 8.98 | 8.29 |
| GLCM_Dissimilarity | 9.35 | 6.51 | 6.75 | 6.77 | 6.79 | 8.99 | 9.05 | 7.19 | 5.47 | 8.58 | 3.18 |
| GLRLM_SRE | 0.972 | 0.971 | 0.958 | 0.971 | 0.966 | 0.977 | 0.978 | 0.96 | 0.965 | 0.973 | 0.91 |
| GLRLM_LRE | 1.13 | 1.13 | 1.19 | 1.14 | 1.16 | 1.1 | 1.1 | 1.19 | 1.16 | 1.12 | 1.5 |
| GLRLM_LGRE | 0.00214 | 0.00357 | 0.00153 | 0.00199 | 0.00183 | 0.00291 | 0.00132 | 0.0041 | 0.00575 | 0.00577 | 0.00259 |
| GLRLM_HGRE | 1750 | 1620 | 1980 | 1560 | 1970 | 1590 | 1760 | 1940 | 1580 | 1950 | 900 |
| GLRLM_SRLGE | 0.0021 | 0.00348 | 0.00151 | 0.00197 | 0.00181 | 0.00287 | 0.00131 | 0.00399 | 0.00565 | 0.00566 | 0.00244 |
| GLRLM_SRHGE | 1680 | 1570 | 1890 | 1510 | 1890 | 1550 | 1710 | 1840 | 1520 | 1890 | 819 |
| GLRLM_LRLGE | 0.00231 | 0.00396 | 0.00163 | 0.00209 | 0.00191 | 0.0031 | 0.00139 | 0.00466 | 0.00771 | 0.00621 | 0.00336 |
| GLRLM_LRHGE | 2050 | 1840 | 2390 | 1780 | 2350 | 1780 | 1970 | 2380 | 1850 | 2230 | 1350 |
| GLRLM_GLNU | 83.4 | 26.9 | 74.5 | 32.2 | 36.6 | 48 | 18 | 92.9 | 48.4 | 23.1 | 602 |
| GLRLM_RLNU | 3250 | 789 | 1510 | 918 | 929 | 2100 | 635 | 2710 | 988 | 772 | 9310 |
| GLRLM_RP | 0.962 | 0.961 | 0.944 | 0.96 | 0.954 | 0.969 | 0.97 | 0.946 | 0.954 | 0.964 | 0.879 |
| NGLDM_Coarseness | 0.0021 | 0.0109 | 0.00348 | 0.00818 | 0.00771 | 0.00396 | 0.0108 | 0.00266 | 0.00701 | 0.00908 | 0.000838 |
| NGLDM_Contrast | 0.562 | 0.242 | 0.212 | 0.255 | 0.237 | 0.494 | 0.432 | 0.444 | 0.169 | 0.564 | 0.0581 |
| NGLDM_Busyness | 0.178 | 0.0417 | 0.152 | 0.0796 | 0.0462 | 0.106 | 0.0444 | 0.107 | 0.0526 | 0.0378 | 0.451 |
| GLZLM_SZE | 0.794 | 0.718 | 0.711 | 0.714 | 0.743 | 0.788 | 0.79 | 0.747 | 0.756 | 0.777 | 0.671 |
| GLZLM_LZE | 5.35 | 4.93 | 12.4 | 4.79 | 8.06 | 3.58 | 3.38 | 18.7 | 8.55 | 5.09 | 862 |
| GLZLM_LGZE | 0.00234 | 0.00353 | 0.00251 | 0.00276 | 0.00278 | 0.00328 | 0.00158 | 0.00437 | 0.00342 | 0.00641 | 0.00411 |

| Patient | 144 | 145 | 146 | 147 | 148 | 149 | 150 | 151 | 152 | 153 | 154 |
| --- | --- | --- | --- | --- | --- | --- | --- | --- | --- | --- | --- |
| Lesion (benign=0,malignant=1) | 1 | 1 | 1 | 1 | 1 | 1 | 1 | 1 | 1 | 1 | 1 |
| minValue | 0 | 141 | 93 | 23 | 81 | 157 | 158 | 15 | 14 | 69 | 10 |
| meanValue | 209 | 439 | 436 | 106 | 284 | 497 | 611 | 127 | 183 | 326 | 107 |
| stdValue | 42.7 | 116 | 108 | 20.7 | 65.3 | 126 | 166 | 30.3 | 28.6 | 125 | 38.2 |
| maxValue | 499 | 665 | 674 | 179 | 405 | 709 | 897 | 184 | 256 | 593 | 202 |
| HISTO_Skewness | 0.155 | -0.317 | -0.927 | -0.441 | -0.536 | -0.457 | -0.55 | -0.822 | -1.2 | -0.127 | -0.478 |
| HISTO_Kurtosis | 7.5 | 2.12 | 3.24 | 4.17 | 2.89 | 2.41 | 2.62 | 3.46 | 5.86 | 1.76 | 2.51 |
| HISTO_Entropy_log2 | 4.24 | 5.56 | 5.32 | 5.04 | 5.48 | 5.52 | 5.6 | 5.39 | 4.78 | 5.71 | 5.58 |
| GLCM_Homogeneity | 0.511 | 0.155 | 0.24 | 0.291 | 0.151 | 0.156 | 0.162 | 0.197 | 0.283 | 0.184 | 0.213 |
| GLCM_Energy | 0.0205 | 0.00326 | 0.0027 | 0.00255 | 0.00441 | 0.00403 | 0.00386 | 0.00188 | 0.00396 | 0.00161 | 0.00105 |
| GLCM_Contrast | 18.5 | 335 | 107 | 55.7 | 231 | 280 | 274 | 144 | 71.7 | 293 | 140 |
| GLCM_Correlation | 0.631 | 0.111 | 0.421 | 0.578 | 0.137 | 0.135 | 0.228 | 0.278 | 0.207 | 0.266 | 0.581 |
| GLCM_Entropy_log2 | 7.37 | 8.41 | 9.03 | 9.41 | 7.99 | 8.1 | 8.16 | 9.37 | 8.75 | 9.58 | 10.3 |
| GLCM_Dissimilarity | 2.49 | 14.4 | 7.5 | 5.37 | 12.4 | 12.9 | 12.8 | 9.19 | 5.91 | 12.7 | 8.83 |
| GLRLM_SRE | 0.862 | 0.981 | 0.968 | 0.952 | 0.984 | 0.985 | 0.984 | 0.977 | 0.959 | 0.979 | 0.972 |
| GLRLM_LRE | 2.35 | 1.08 | 1.14 | 1.23 | 1.07 | 1.06 | 1.07 | 1.1 | 1.2 | 1.1 | 1.12 |
| GLRLM_LGRE | 0.00263 | 0.00474 | 0.00315 | 0.00197 | 0.00581 | 0.00162 | 0.00605 | 0.0028 | 0.000895 | 0.00519 | 0.00337 |
| GLRLM_HGRE | 778 | 1550 | 1600 | 1260 | 1820 | 1870 | 1770 | 1960 | 2080 | 1240 | 1250 |
| GLRLM_SRLGE | 0.00239 | 0.00472 | 0.00311 | 0.0019 | 0.0058 | 0.00161 | 0.00604 | 0.00274 | 0.000875 | 0.00511 | 0.00324 |
| GLRLM_SRHGE | 673 | 1510 | 1530 | 1200 | 1780 | 1830 | 1730 | 1900 | 1990 | 1210 | 1210 |
| GLRLM_LRLGE | 0.00478 | 0.00482 | 0.00331 | 0.00229 | 0.00585 | 0.00166 | 0.00608 | 0.00307 | 0.00099 | 0.00549 | 0.00395 |
| GLRLM_LRHGE | 1800 | 1710 | 1870 | 1560 | 1980 | 2020 | 1930 | 2200 | 2530 | 1390 | 1400 |
| GLRLM_GLNU | 700 | 8.19 | 32.6 | 432 | 7.84 | 7.14 | 7.66 | 32.2 | 160 | 23 | 92.4 |
| GLRLM_RLNU | 7610 | 325 | 941 | 10000 | 288 | 277 | 309 | 1080 | 3040 | 1040 | 3610 |
| GLRLM_RP | 0.789 | 0.975 | 0.957 | 0.936 | 0.978 | 0.98 | 0.979 | 0.969 | 0.944 | 0.971 | 0.963 |
| NGLDM_Coarseness | 0.000867 | 0.0121 | 0.00755 | 0.000786 | 0.0176 | 0.0171 | 0.0176 | 0.00575 | 0.00215 | 0.00548 | 0.00209 |
| NGLDM_Contrast | 0.0278 | 1.27 | 0.376 | 0.122 | 0.838 | 1.02 | 0.911 | 0.428 | 0.122 | 0.958 | 0.439 |
| NGLDM_Busyness | 0.44 | 0.0461 | 0.0449 | 0.447 | 0.032 | 0.031 | 0.026 | 0.0618 | 0.139 | 0.099 | 0.213 |
| GLZLM_SZE | 0.679 | 0.842 | 0.757 | 0.674 | 0.835 | 0.84 | 0.832 | 0.796 | 0.728 | 0.812 | 0.769 |
| GLZLM_LZE | 1790 | 2.88 | 7.78 | 19.9 | 2.53 | 2.36 | 2.45 | 3.93 | 15.6 | 3.55 | 5.5 |
| GLZLM_LGZE | 0.00281 | 0.00597 | 0.00431 | 0.00257 | 0.00734 | 0.00192 | 0.00765 | 0.00266 | 0.00125 | 0.00582 | 0.00298 |

| Patient | 155 | 156 | 157 | 158 | 159 | 160 | 161 | 162 | 163 | 164 | 165 |
| --- | --- | --- | --- | --- | --- | --- | --- | --- | --- | --- | --- |
| Lesion (benign=0,malignant=1) | 1 | 1 | 1 | 1 | 1 | 1 | 1 | 1 | 1 | 1 | 1 |
| minValue | 38 | 63 | 35 | 35 | 18 | 23 | 19 | 33 | 23 | 26 | 8 |
| meanValue | 228 | 483 | 219 | 440 | 200 | 170 | 134 | 306 | 357 | 126 | 165 |
| stdValue | 70.9 | 143 | 53 | 152 | 57.6 | 53.2 | 40.4 | 51.9 | 96.1 | 25.8 | 38.6 |
| maxValue | 350 | 693 | 319 | 732 | 367 | 283 | 245 | 440 | 579 | 187 | 251 |
| HISTO_Skewness | -0.808 | -0.947 | -0.904 | -0.371 | -0.661 | -0.818 | 0.0701 | -1.02 | -0.279 | -0.515 | -0.496 |
| HISTO_Kurtosis | 2.79 | 3 | 3.56 | 2.1 | 3.13 | 2.94 | 2.44 | 4.81 | 2.56 | 3.07 | 3.66 |
| HISTO_Entropy_log2 | 5.57 | 5.58 | 5.43 | 5.68 | 5.33 | 5.52 | 5.51 | 4.94 | 5.43 | 5.32 | 5.32 |
| GLCM_Homogeneity | 0.185 | 0.245 | 0.261 | 0.208 | 0.272 | 0.226 | 0.193 | 0.282 | 0.194 | 0.222 | 0.221 |
| GLCM_Energy | 0.003 | 0.00184 | 0.00209 | 0.00129 | 0.00197 | 0.00171 | 0.00124 | 0.00283 | 0.00183 | 0.00135 | 0.00166 |
| GLCM_Contrast | 340 | 114 | 114 | 183 | 84.7 | 147 | 158 | 61.1 | 149 | 104 | 120 |
| GLCM_Correlation | 0.0403 | 0.602 | 0.513 | 0.412 | 0.516 | 0.494 | 0.362 | 0.481 | 0.351 | 0.464 | 0.314 |
| GLCM_Entropy_log2 | 8.57 | 9.62 | 9.4 | 10 | 9.82 | 9.72 | 9.96 | 8.97 | 9.33 | 9.93 | 9.56 |
| GLCM_Dissimilarity | 13.2 | 7.46 | 7.42 | 9.87 | 6.43 | 8.62 | 9.65 | 5.53 | 9.44 | 7.73 | 8.22 |
| GLRLM_SRE | 0.979 | 0.968 | 0.96 | 0.976 | 0.958 | 0.972 | 0.978 | 0.961 | 0.979 | 0.97 | 0.972 |
| GLRLM_LRE | 1.1 | 1.15 | 1.19 | 1.11 | 1.2 | 1.13 | 1.1 | 1.19 | 1.09 | 1.13 | 1.13 |
| GLRLM_LGRE | 0.00662 | 0.00375 | 0.00432 | 0.00251 | 0.0025 | 0.0062 | 0.00251 | 0.00112 | 0.0023 | 0.00164 | 0.00252 |
| GLRLM_HGRE | 1760 | 2050 | 1880 | 1600 | 1250 | 1500 | 1210 | 1950 | 1630 | 1720 | 1840 |
| GLRLM_SRLGE | 0.00656 | 0.00371 | 0.00425 | 0.00248 | 0.00243 | 0.00609 | 0.00245 | 0.0011 | 0.00228 | 0.00162 | 0.00246 |
| GLRLM_SRHGE | 1710 | 1960 | 1790 | 1550 | 1190 | 1450 | 1190 | 1870 | 1590 | 1670 | 1780 |
| GLRLM_LRLGE | 0.00688 | 0.00394 | 0.00519 | 0.00262 | 0.00284 | 0.00663 | 0.00275 | 0.00123 | 0.00236 | 0.00174 | 0.00291 |
| GLRLM_LRHGE | 1980 | 2440 | 2290 | 1830 | 1530 | 1720 | 1340 | 2350 | 1820 | 1960 | 2120 |
| GLRLM_GLNU | 10.5 | 53.8 | 44.7 | 55.3 | 308 | 52 | 50.9 | 89.2 | 21.1 | 86.2 | 50.1 |
| GLRLM_RLNU | 407 | 2010 | 1480 | 2450 | 9270 | 1860 | 1950 | 2050 | 771 | 2740 | 1620 |
| GLRLM_RP | 0.971 | 0.957 | 0.947 | 0.967 | 0.944 | 0.962 | 0.971 | 0.947 | 0.971 | 0.96 | 0.962 |
| NGLDM_Coarseness | 0.0123 | 0.00454 | 0.00477 | 0.00304 | 0.000852 | 0.00408 | 0.00353 | 0.00356 | 0.00824 | 0.00262 | 0.0039 |
| NGLDM_Contrast | 1.02 | 0.474 | 0.37 | 0.638 | 0.265 | 0.491 | 0.42 | 0.143 | 0.476 | 0.293 | 0.312 |
| NGLDM_Busyness | 0.0303 | 0.0684 | 0.0679 | 0.154 | 0.479 | 0.0943 | 0.168 | 0.0953 | 0.0675 | 0.153 | 0.1 |
| GLZLM_SZE | 0.827 | 0.776 | 0.719 | 0.786 | 0.726 | 0.762 | 0.778 | 0.711 | 0.791 | 0.749 | 0.756 |
| GLZLM_LZE | 3.29 | 7.89 | 9.42 | 4.49 | 23.8 | 5.73 | 3.23 | 10.1 | 3.22 | 5.77 | 4.71 |
| GLZLM_LGZE | 0.00787 | 0.00509 | 0.00418 | 0.00315 | 0.00301 | 0.00727 | 0.00239 | 0.0016 | 0.00294 | 0.0022 | 0.00215 |

| Patient | 166 | 167 | 168 | 169 | 170 | 171 | 172 | 173 | 174 | 175 | 176 |
| --- | --- | --- | --- | --- | --- | --- | --- | --- | --- | --- | --- |
| Lesion (benign=0,malignant=1) | 1 | 1 | 1 | 1 | 1 | 1 | 1 | 1 | 1 | 1 | 1 |
| minValue | 264 | 77 | 39 | 308 | 43 | 64 | 178 | 288 | 42 | 24 | 44 |
| meanValue | 535 | 351 | 152 | 418 | 71.1 | 270 | 323 | 436 | 83.7 | 293 | 155 |
| stdValue | 86.9 | 112 | 35.3 | 52 | 6.08 | 58.4 | 38.6 | 58.7 | 17 | 67.6 | 54.3 |
| maxValue | 699 | 588 | 234 | 566 | 88 | 439 | 456 | 543 | 132 | 429 | 321 |
| HISTO_Skewness | -0.939 | -0.493 | -0.575 | 0.475 | -0.753 | -0.55 | 0.217 | -0.706 | 0.371 | -0.812 | 0.316 |
| HISTO_Kurtosis | 3.35 | 2.23 | 3.21 | 3.41 | 4.44 | 3.16 | 2.99 | 2.63 | 2.58 | 3.26 | 2.43 |
| HISTO_Entropy_log2 | 5.43 | 5.56 | 5.47 | 5.22 | 4.57 | 5.3 | 5.1 | 5.6 | 5.42 | 5.27 | 5.6 |
| GLCM_Homogeneity | 0.184 | 0.199 | 0.193 | 0.178 | 0.244 | 0.257 | 0.238 | 0.184 | 0.186 | 0.22 | 0.222 |
| GLCM_Energy | 0.0023 | 0.00256 | 0.00188 | 0.00815 | 0.00386 | 0.00174 | 0.00232 | 0.00358 | 0.00252 | 0.00253 | 0.00114 |
| GLCM_Contrast | 236 | 223 | 187 | 201 | 82.8 | 76 | 84.3 | 355 | 126 | 150 | 117 |
| GLCM_Correlation | 0.41 | 0.285 | 0.272 | 0.353 | 0.377 | 0.532 | 0.408 | 0.17 | 0.52 | 0.173 | 0.606 |
| GLCM_Entropy_log2 | 8.96 | 8.85 | 9.4 | 7.05 | 8.43 | 9.67 | 9.19 | 8.27 | 8.84 | 8.98 | 10.1 |
| GLCM_Dissimilarity | 11.5 | 11.1 | 10.3 | 11.2 | 6.96 | 6.38 | 6.92 | 13.7 | 8.98 | 9.02 | 8.11 |
| GLRLM_SRE | 0.978 | 0.976 | 0.979 | 0.981 | 0.959 | 0.962 | 0.969 | 0.978 | 0.982 | 0.973 | 0.97 |
| GLRLM_LRE | 1.09 | 1.11 | 1.09 | 1.08 | 1.19 | 1.17 | 1.14 | 1.09 | 1.08 | 1.13 | 1.13 |
| GLRLM_LGRE | 0.00775 | 0.00894 | 0.00474 | 0.0281 | 0.00179 | 0.00187 | 0.00197 | 0.00855 | 0.00391 | 0.00196 | 0.00516 |
| GLRLM_HGRE | 1790 | 1400 | 1550 | 945 | 1710 | 1360 | 1240 | 1620 | 1060 | 1950 | 852 |
| GLRLM_SRLGE | 0.00761 | 0.00881 | 0.00467 | 0.0255 | 0.00176 | 0.00184 | 0.00193 | 0.00845 | 0.00387 | 0.00194 | 0.00499 |
| GLRLM_SRHGE | 1750 | 1350 | 1510 | 935 | 1640 | 1300 | 1200 | 1580 | 1050 | 1890 | 831 |
| GLRLM_LRLGE | 0.00831 | 0.00948 | 0.00507 | 0.0384 | 0.00192 | 0.00202 | 0.00211 | 0.00897 | 0.00407 | 0.00202 | 0.00593 |
| GLRLM_LRHGE | 1970 | 1590 | 1710 | 985 | 2030 | 1630 | 1390 | 1790 | 1130 | 2260 | 942 |
| GLRLM_GLNU | 14.4 | 13.2 | 27.7 | 4.38 | 57.6 | 101 | 43.1 | 7.79 | 16.1 | 26.1 | 60.9 |
| GLRLM_RLNU | 483 | 503 | 973 | 132 | 1020 | 3110 | 1160 | 298 | 538 | 802 | 2480 |
| GLRLM_RP | 0.971 | 0.967 | 0.972 | 0.976 | 0.946 | 0.95 | 0.958 | 0.972 | 0.976 | 0.963 | 0.96 |
| NGLDM_Coarseness | 0.0107 | 0.0102 | 0.00639 | 0.0398 | 0.00431 | 0.00258 | 0.00559 | 0.0167 | 0.0122 | 0.00692 | 0.00311 |
| NGLDM_Contrast | 0.659 | 0.874 | 0.474 | 0.745 | 0.198 | 0.221 | 0.255 | 1.01 | 0.463 | 0.479 | 0.408 |
| NGLDM_Busyness | 0.0354 | 0.0439 | 0.0634 | 0.0305 | 0.174 | 0.145 | 0.109 | 0.0231 | 0.0553 | 0.0633 | 0.226 |
| GLZLM_SZE | 0.779 | 0.825 | 0.808 | 0.821 | 0.687 | 0.733 | 0.728 | 0.786 | 0.795 | 0.791 | 0.747 |
| GLZLM_LZE | 3.05 | 4.1 | 3.28 | 2.63 | 10.6 | 11.7 | 6.45 | 3.32 | 2.58 | 5.11 | 5.45 |
| GLZLM_LGZE | 0.00799 | 0.00982 | 0.00493 | 0.0123 | 0.00277 | 0.00267 | 0.00255 | 0.01 | 0.00453 | 0.00266 | 0.005 |

| Patient | 177 | 178 | 179 | 180 | 181 | 182 | 183 | 184 | 185 | 186 | 187 |
| --- | --- | --- | --- | --- | --- | --- | --- | --- | --- | --- | --- |
| Lesion (benign=0,malignant=1) | 1 | 1 | 1 | 1 | 1 | 1 | 1 | 1 | 1 | 1 | 1 |
| minValue | 178 | 111 | 58 | 27 | 21 | 91 | 57 | 135 | 176 | 75 | 27 |
| meanValue | 352 | 450 | 188 | 215 | 60.7 | 405 | 87 | 418 | 441 | 256 | 70.9 |
| stdValue | 54.1 | 92.3 | 36.5 | 65.2 | 10.5 | 107 | 11.6 | 100 | 60.9 | 53.3 | 15 |
| maxValue | 482 | 708 | 318 | 494 | 83 | 649 | 130 | 633 | 710 | 422 | 106 |
| HISTO_Skewness | -0.647 | 0.113 | -0.331 | 0.593 | -0.755 | -0.37 | 0.52 | -0.39 | 0.158 | -0.249 | 0.346 |
| HISTO_Kurtosis | 3.21 | 3.41 | 3.08 | 3.84 | 3.27 | 2.77 | 3.09 | 2.67 | 3.58 | 2.86 | 3.02 |
| HISTO_Entropy_log2 | 5.33 | 5.25 | 5.17 | 5.14 | 5.26 | 5.5 | 5.21 | 5.54 | 4.86 | 5.29 | 5.36 |
| GLCM_Homogeneity | 0.197 | 0.253 | 0.216 | 0.259 | 0.199 | 0.185 | 0.212 | 0.161 | 0.274 | 0.271 | 0.149 |
| GLCM_Energy | 0.00488 | 0.00275 | 0.00177 | 0.00195 | 0.00277 | 0.00348 | 0.00208 | 0.00363 | 0.00265 | 0.00205 | 0.00408 |
| GLCM_Contrast | 157 | 71.9 | 109 | 66.4 | 129 | 179 | 124 | 226 | 61 | 66.7 | 308 |
| GLCM_Correlation | 0.373 | 0.624 | 0.25 | 0.512 | 0.425 | 0.301 | 0.418 | 0.22 | 0.392 | 0.645 | 0.117 |
| GLCM_Entropy_log2 | 7.86 | 8.93 | 9.52 | 9.59 | 8.74 | 8.33 | 9.24 | 8.22 | 9.23 | 9.37 | 8.04 |
| GLCM_Dissimilarity | 9.49 | 6.44 | 7.97 | 6.11 | 8.82 | 10.3 | 8.53 | 11.9 | 5.77 | 5.93 | 13.7 |
| GLRLM_SRE | 0.983 | 0.966 | 0.976 | 0.96 | 0.979 | 0.98 | 0.971 | 0.985 | 0.956 | 0.97 | 0.987 |
| GLRLM_LRE | 1.07 | 1.16 | 1.11 | 1.18 | 1.1 | 1.08 | 1.12 | 1.06 | 1.21 | 1.14 | 1.05 |
| GLRLM_LGRE | 0.00468 | 0.00245 | 0.00304 | 0.00316 | 0.00246 | 0.00697 | 0.00343 | 0.00522 | 0.00172 | 0.00273 | 0.00513 |
| GLRLM_HGRE | 1510 | 1460 | 1130 | 774 | 1830 | 1480 | 826 | 1520 | 1100 | 1240 | 1450 |
| GLRLM_SRLGE | 0.00467 | 0.00242 | 0.00302 | 0.00308 | 0.00245 | 0.00695 | 0.00335 | 0.00521 | 0.00164 | 0.0027 | 0.00512 |
| GLRLM_SRHGE | 1480 | 1410 | 1100 | 745 | 1790 | 1440 | 806 | 1490 | 1050 | 1200 | 1430 |
| GLRLM_LRLGE | 0.00474 | 0.00258 | 0.00314 | 0.00351 | 0.00252 | 0.00704 | 0.00374 | 0.00528 | 0.00208 | 0.00288 | 0.00519 |
| GLRLM_LRHGE | 1630 | 1670 | 1260 | 908 | 2060 | 1620 | 911 | 1640 | 1310 | 1420 | 1520 |
| GLRLM_GLNU | 9.63 | 28.1 | 64.6 | 189 | 20.1 | 9.69 | 31.2 | 7.49 | 244 | 72 | 7.29 |
| GLRLM_RLNU | 312 | 796 | 1880 | 4940 | 627 | 364 | 887 | 291 | 5300 | 2260 | 243 |
| GLRLM_RP | 0.978 | 0.954 | 0.967 | 0.947 | 0.971 | 0.975 | 0.962 | 0.981 | 0.941 | 0.96 | 0.983 |
| NGLDM_Coarseness | 0.0222 | 0.00963 | 0.00342 | 0.00156 | 0.00959 | 0.0173 | 0.00689 | 0.0181 | 0.00117 | 0.00433 | 0.0181 |
| NGLDM_Contrast | 0.492 | 0.223 | 0.277 | 0.174 | 0.458 | 0.602 | 0.347 | 0.772 | 0.129 | 0.18 | 0.886 |
| NGLDM_Busyness | 0.0249 | 0.0515 | 0.15 | 0.365 | 0.0542 | 0.0321 | 0.112 | 0.0298 | 0.383 | 0.102 | 0.0449 |
| GLZLM_SZE | 0.837 | 0.725 | 0.77 | 0.687 | 0.786 | 0.813 | 0.737 | 0.847 | 0.692 | 0.75 | 0.837 |
| GLZLM_LZE | 2.41 | 7.12 | 4.18 | 14 | 3.23 | 2.64 | 4.63 | 2.2 | 31.1 | 5.37 | 1.94 |
| GLZLM_LGZE | 0.00584 | 0.00361 | 0.00401 | 0.00426 | 0.00323 | 0.00903 | 0.00403 | 0.00635 | 0.00175 | 0.00373 | 0.00614 |

| Patient | 188 | 189 | 190 | 191 | 192 | 193 | 194 | 195 | 196 | 197 | 198 |
| --- | --- | --- | --- | --- | --- | --- | --- | --- | --- | --- | --- |
| Lesion (benign=0,malignant=1) | 1 | 1 | 1 | 1 | 1 | 1 | 1 | 1 | 1 | 1 | 1 |
| minValue | 23 | 36 | 27 | 53 | 51 | 25 | 142 | 104 | 323 | 179 | 13 |
| meanValue | 128 | 163 | 227 | 194 | 134 | 77.8 | 251 | 443 | 492 | 419 | 97.8 |
| stdValue | 33.4 | 33.7 | 42.1 | 37.3 | 27.3 | 16 | 32.5 | 87.5 | 40.1 | 95.1 | 20.1 |
| maxValue | 231 | 249 | 331 | 273 | 180 | 116 | 313 | 675 | 560 | 594 | 167 |
| HISTO_Skewness | -0.294 | -1.19 | -0.932 | -0.583 | -0.798 | -0.64 | -0.556 | -0.521 | -1.37 | -0.26 | -0.845 |
| HISTO_Kurtosis | 2.8 | 4.57 | 4.12 | 3.29 | 2.98 | 3.35 | 3.12 | 3.3 | 5.25 | 2.34 | 4.35 |
| HISTO_Entropy_log2 | 5.36 | 5.1 | 5.07 | 5.36 | 5.58 | 5.37 | 5.35 | 5.29 | 5.14 | 5.59 | 4.96 |
| GLCM_Homogeneity | 0.221 | 0.246 | 0.276 | 0.199 | 0.217 | 0.199 | 0.182 | 0.225 | 0.235 | 0.14 | 0.283 |
| GLCM_Energy | 0.00133 | 0.00345 | 0.00224 | 0.00254 | 0.00186 | 0.0016 | 0.00419 | 0.00152 | 0.00513 | 0.00855 | 0.00271 |
| GLCM_Contrast | 107 | 96 | 73.3 | 137 | 176 | 164 | 152 | 100 | 91.2 | 293 | 70.9 |
| GLCM_Correlation | 0.461 | 0.399 | 0.43 | 0.27 | 0.401 | 0.367 | 0.421 | 0.411 | 0.368 | 0.174 | 0.48 |
| GLCM_Entropy_log2 | 10.1 | 8.66 | 9.42 | 8.84 | 9.41 | 9.73 | 7.99 | 9.77 | 7.87 | 7.01 | 9.46 |
| GLCM_Dissimilarity | 7.87 | 7.16 | 6.08 | 8.95 | 9.58 | 9.6 | 9.7 | 7.64 | 7.06 | 13.9 | 5.9 |
| GLRLM_SRE | 0.97 | 0.968 | 0.953 | 0.979 | 0.972 | 0.973 | 0.978 | 0.968 | 0.98 | 0.992 | 0.948 |
| GLRLM_LRE | 1.14 | 1.15 | 1.22 | 1.09 | 1.13 | 1.12 | 1.09 | 1.14 | 1.08 | 1.03 | 1.25 |
| GLRLM_LGRE | 0.00207 | 0.0037 | 0.000942 | 0.00273 | 0.00512 | 0.0039 | 0.00708 | 0.00151 | 0.00355 | 0.00824 | 0.00149 |
| GLRLM_HGRE | 1190 | 1600 | 1880 | 1840 | 1890 | 1540 | 1820 | 1570 | 2230 | 1620 | 1340 |
| GLRLM_SRLGE | 0.00204 | 0.00362 | 0.000917 | 0.00271 | 0.0051 | 0.00383 | 0.00707 | 0.00149 | 0.00355 | 0.00823 | 0.00144 |
| GLRLM_SRHGE | 1150 | 1540 | 1780 | 1800 | 1830 | 1500 | 1770 | 1520 | 2180 | 1600 | 1270 |
| GLRLM_LRLGE | 0.00223 | 0.00404 | 0.00106 | 0.00278 | 0.00519 | 0.0042 | 0.00712 | 0.00161 | 0.00359 | 0.00828 | 0.00175 |
| GLRLM_LRHGE | 1370 | 1860 | 2350 | 2030 | 2210 | 1720 | 2080 | 1820 | 2440 | 1700 | 1690 |
| GLRLM_GLNU | 118 | 36.1 | 226 | 15.9 | 23.3 | 45.3 | 4.53 | 71.7 | 12.8 | 3.76 | 800 |
| GLRLM_RLNU | 3850 | 880 | 5720 | 526 | 905 | 1410 | 155 | 2190 | 346 | 156 | 17000 |
| GLRLM_RP | 0.959 | 0.957 | 0.937 | 0.972 | 0.962 | 0.965 | 0.97 | 0.957 | 0.974 | 0.989 | 0.931 |
| NGLDM_Coarseness | 0.0018 | 0.00749 | 0.00121 | 0.0125 | 0.00723 | 0.00397 | 0.0393 | 0.00328 | 0.0199 | 0.0307 | 0.000369 |
| NGLDM_Contrast | 0.295 | 0.284 | 0.187 | 0.438 | 0.64 | 0.412 | 0.666 | 0.26 | 0.367 | 1.1 | 0.141 |
| NGLDM_Busyness | 0.242 | 0.0467 | 0.265 | 0.0381 | 0.0491 | 0.0902 | 0.0176 | 0.119 | 0.0221 | 0.0177 | 0.873 |
| GLZLM_SZE | 0.751 | 0.776 | 0.71 | 0.796 | 0.811 | 0.753 | 0.917 | 0.736 | 0.818 | 0.899 | 0.679 |
| GLZLM_LZE | 6.16 | 6.86 | 20.8 | 3.15 | 6.76 | 4.72 | 1.42 | 6.13 | 3.04 | 1.63 | 49.4 |
| GLZLM_LGZE | 0.00258 | 0.004 | 0.00133 | 0.00358 | 0.00747 | 0.00427 | 0.0077 | 0.00203 | 0.00471 | 0.00929 | 0.00194 |

| Patient | 199 | 200 | 201 | 202 | 203 | 204 | 205 | 206 | 207 | 208 | 209 |
| --- | --- | --- | --- | --- | --- | --- | --- | --- | --- | --- | --- |
| Lesion (benign=0,malignant=1) | 1 | 1 | 1 | 1 | 1 | 1 | 1 | 1 | 1 | 1 | 1 |
| minValue | 36 | 6 | 12 | 12 | 21 | 210 | 105 | 42 | 118 | 88 | 118 |
| meanValue | 58.2 | 156 | 105 | 97.8 | 208 | 386 | 333 | 163 | 317 | 227 | 384 |
| stdValue | 7.68 | 43.4 | 34.7 | 27.6 | 43.6 | 38.7 | 63.6 | 36.4 | 58.3 | 47.9 | 74.3 |
| maxValue | 81 | 257 | 229 | 151 | 315 | 468 | 505 | 247 | 502 | 314 | 517 |
| HISTO_Skewness | 0.476 | -0.701 | 0.233 | -0.636 | -0.952 | -0.962 | -0.673 | -0.601 | -0.0583 | -0.512 | -1.11 |
| HISTO_Kurtosis | 3.38 | 3.13 | 2.89 | 2.69 | 3.94 | 5.2 | 3.73 | 2.94 | 3 | 2.31 | 3.72 |
| HISTO_Entropy_log2 | 4.84 | 5.41 | 5.37 | 5.48 | 5.15 | 4.93 | 5.29 | 5.46 | 5.24 | 5.48 | 5.29 |
| GLCM_Homogeneity | 0.193 | 0.229 | 0.265 | 0.216 | 0.279 | 0.193 | 0.198 | 0.196 | 0.197 | 0.192 | 0.226 |
| GLCM_Energy | 0.00413 | 0.00126 | 0.00153 | 0.00236 | 0.00216 | 0.00903 | 0.00196 | 0.0011 | 0.00289 | 0.00264 | 0.00236 |
| GLCM_Contrast | 166 | 118 | 59.6 | 192 | 69.3 | 134 | 162 | 165 | 155 | 261 | 133 |
| GLCM_Correlation | 0.296 | 0.459 | 0.692 | 0.238 | 0.557 | 0.223 | 0.256 | 0.4 | 0.142 | 0.179 | 0.389 |
| GLCM_Entropy_log2 | 8.16 | 10.2 | 9.87 | 9.16 | 9.63 | 6.92 | 9.33 | 10.3 | 8.67 | 9.01 | 9.24 |
| GLCM_Dissimilarity | 9.95 | 8.02 | 5.83 | 10.2 | 5.94 | 9.07 | 9.71 | 9.67 | 9.59 | 12.2 | 8.36 |
| GLRLM_SRE | 0.975 | 0.968 | 0.96 | 0.968 | 0.953 | 0.983 | 0.977 | 0.977 | 0.98 | 0.975 | 0.97 |
| GLRLM_LRE | 1.11 | 1.15 | 1.18 | 1.16 | 1.22 | 1.07 | 1.11 | 1.1 | 1.09 | 1.12 | 1.14 |
| GLRLM_LGRE | 0.00431 | 0.00152 | 0.00325 | 0.00266 | 0.0012 | 0.00766 | 0.00404 | 0.0016 | 0.00283 | 0.0045 | 0.00314 |
| GLRLM_HGRE | 1150 | 1600 | 892 | 1740 | 1780 | 2040 | 1470 | 1590 | 1230 | 1750 | 1990 |
| GLRLM_SRLGE | 0.00427 | 0.00148 | 0.00316 | 0.00264 | 0.00117 | 0.00765 | 0.00397 | 0.00158 | 0.00282 | 0.00448 | 0.00312 |
| GLRLM_SRHGE | 1130 | 1540 | 855 | 1660 | 1680 | 2010 | 1430 | 1550 | 1200 | 1690 | 1910 |
| GLRLM_LRLGE | 0.00452 | 0.00169 | 0.00368 | 0.00275 | 0.00136 | 0.0077 | 0.00432 | 0.00169 | 0.00292 | 0.00455 | 0.0032 |
| GLRLM_LRHGE | 1260 | 1880 | 1060 | 2090 | 2230 | 2180 | 1640 | 1770 | 1340 | 2070 | 2320 |
| GLRLM_GLNU | 18.5 | 294 | 154 | 22.9 | 567 | 5.53 | 36.1 | 89.5 | 20.1 | 19.8 | 40.8 |
| GLRLM_RLNU | 407 | 9870 | 4940 | 795 | 14700 | 135 | 1110 | 3210 | 626 | 704 | 1190 |
| GLRLM_RP | 0.967 | 0.957 | 0.947 | 0.955 | 0.937 | 0.977 | 0.968 | 0.97 | 0.974 | 0.965 | 0.959 |
| NGLDM_Coarseness | 0.0106 | 0.0007 | 0.00188 | 0.00619 | 0.000504 | 0.0375 | 0.00487 | 0.00204 | 0.00938 | 0.00582 | 0.00495 |
| NGLDM_Contrast | 0.391 | 0.324 | 0.192 | 0.655 | 0.177 | 0.55 | 0.349 | 0.425 | 0.366 | 0.852 | 0.454 |
| NGLDM_Busyness | 0.101 | 0.496 | 0.287 | 0.0592 | 0.573 | 0.0333 | 0.0731 | 0.197 | 0.0665 | 0.0603 | 0.0621 |
| GLZLM_SZE | 0.746 | 0.746 | 0.707 | 0.781 | 0.723 | 0.81 | 0.799 | 0.787 | 0.785 | 0.83 | 0.774 |
| GLZLM_LZE | 4.09 | 7.25 | 12.4 | 9.18 | 27.5 | 2.52 | 4.03 | 3.64 | 2.87 | 5.3 | 7.68 |
| GLZLM_LGZE | 0.00558 | 0.00175 | 0.00395 | 0.00399 | 0.00168 | 0.01 | 0.00436 | 0.00184 | 0.00353 | 0.00617 | 0.00476 |

| Patient | 210 | 211 | 212 | 213 | 214 | 215 | 216 | 217 | 218 | 219 | 220 |
| --- | --- | --- | --- | --- | --- | --- | --- | --- | --- | --- | --- |
| Lesion (benign=0,malignant=1) | 1 | 1 | 1 | 1 | 1 | 1 | 1 | 1 | 1 | 1 | 1 |
| minValue | 29 | 411 | 32 | 330 | 26 | 87 | 154 | 8 | 78 | 34 | 24 |
| meanValue | 65.4 | 512 | 130 | 445 | 187 | 187 | 325 | 80.4 | 399 | 185 | 151 |
| stdValue | 12 | 30.6 | 23.9 | 35.2 | 37.5 | 25.2 | 47.3 | 19.5 | 93.9 | 61.7 | 37.5 |
| maxValue | 92 | 583 | 214 | 539 | 294 | 321 | 465 | 168 | 577 | 295 | 252 |
| HISTO_Skewness | -0.465 | -0.55 | -0.212 | -0.113 | -0.842 | 0.374 | -0.182 | -0.158 | -0.774 | -0.171 | -0.473 |
| HISTO_Kurtosis | 2.62 | 3.23 | 3.54 | 2.95 | 3.57 | 4.74 | 2.68 | 3.56 | 3.09 | 2.09 | 3.07 |
| HISTO_Entropy_log2 | 5.47 | 5.35 | 5.07 | 5.36 | 5.09 | 4.75 | 5.3 | 4.94 | 5.46 | 5.68 | 5.37 |
| GLCM_Homogeneity | 0.169 | 0.199 | 0.233 | 0.179 | 0.299 | 0.33 | 0.223 | 0.313 | 0.211 | 0.151 | 0.228 |
| GLCM_Energy | 0.00279 | 0.00363 | 0.00187 | 0.00343 | 0.00245 | 0.00378 | 0.00142 | 0.00283 | 0.00163 | 0.00291 | 0.00172 |
| GLCM_Contrast | 239 | 167 | 99.9 | 165 | 54 | 36.7 | 93.9 | 48.1 | 198 | 379 | 125 |
| GLCM_Correlation | 0.0976 | 0.282 | 0.238 | 0.261 | 0.616 | 0.575 | 0.5 | 0.581 | 0.15 | 0.0986 | 0.32 |
| GLCM_Entropy_log2 | 8.65 | 8.26 | 9.68 | 8.32 | 9.43 | 8.87 | 9.77 | 9.29 | 9.67 | 8.57 | 9.76 |
| GLCM_Dissimilarity | 12 | 9.63 | 7.47 | 9.99 | 5.26 | 4.28 | 7.45 | 4.88 | 9.97 | 15.4 | 8.23 |
| GLRLM_SRE | 0.984 | 0.979 | 0.967 | 0.985 | 0.946 | 0.936 | 0.972 | 0.938 | 0.973 | 0.984 | 0.969 |
| GLRLM_LRE | 1.07 | 1.09 | 1.15 | 1.06 | 1.27 | 1.31 | 1.12 | 1.31 | 1.12 | 1.07 | 1.15 |
| GLRLM_LGRE | 0.00379 | 0.00465 | 0.00136 | 0.00404 | 0.00103 | 0.00234 | 0.00181 | 0.00169 | 0.00215 | 0.00865 | 0.00177 |
| GLRLM_HGRE | 1540 | 1560 | 1280 | 1390 | 1580 | 827 | 1360 | 928 | 1880 | 1620 | 1410 |
| GLRLM_SRLGE | 0.00378 | 0.00463 | 0.00133 | 0.00402 | 0.000993 | 0.00223 | 0.00178 | 0.00161 | 0.00209 | 0.00864 | 0.00174 |
| GLRLM_SRHGE | 1510 | 1530 | 1240 | 1370 | 1480 | 776 | 1320 | 866 | 1820 | 1590 | 1360 |
| GLRLM_LRLGE | 0.00385 | 0.00471 | 0.0015 | 0.0041 | 0.0012 | 0.00285 | 0.00194 | 0.00211 | 0.00236 | 0.0087 | 0.00188 |
| GLRLM_LRHGE | 1660 | 1720 | 1480 | 1470 | 2060 | 1070 | 1530 | 1240 | 2130 | 1790 | 1670 |
| GLRLM_GLNU | 11.8 | 10.5 | 197 | 10.1 | 407 | 511 | 52.7 | 2710 | 46.8 | 8.87 | 96 |
| GLRLM_RLNU | 447 | 336 | 4970 | 347 | 10200 | 9310 | 1710 | 58900 | 1630 | 388 | 3100 |
| GLRLM_RP | 0.979 | 0.972 | 0.955 | 0.98 | 0.927 | 0.914 | 0.962 | 0.917 | 0.965 | 0.978 | 0.957 |
| NGLDM_Coarseness | 0.0112 | 0.0174 | 0.00125 | 0.017 | 0.000784 | 0.000786 | 0.0044 | 0.000113 | 0.00384 | 0.0108 | 0.00203 |
| NGLDM_Contrast | 0.734 | 0.513 | 0.185 | 0.522 | 0.153 | 0.071 | 0.282 | 0.106 | 0.55 | 1.22 | 0.33 |
| NGLDM_Busyness | 0.0456 | 0.0278 | 0.286 | 0.0416 | 0.437 | 0.531 | 0.126 | 3.51 | 0.0996 | 0.0434 | 0.192 |
| GLZLM_SZE | 0.828 | 0.826 | 0.727 | 0.821 | 0.705 | 0.682 | 0.737 | 0.664 | 0.774 | 0.836 | 0.777 |
| GLZLM_LZE | 2.34 | 2.74 | 8.31 | 2.27 | 61.1 | 150 | 4.6 | 353 | 4.43 | 2.8 | 8.02 |
| GLZLM_LGZE | 0.00465 | 0.00595 | 0.00168 | 0.00494 | 0.00145 | 0.0032 | 0.00228 | 0.00212 | 0.0021 | 0.0111 | 0.00234 |

| Patient | 221 | 222 | 223 | 224 | 225 | 226 | 227 | 228 | 229 | 230 | 231 |
| --- | --- | --- | --- | --- | --- | --- | --- | --- | --- | --- | --- |
| Lesion (benign=0,malignant=1) | 1 | 1 | 1 | 1 | 1 | 1 | 1 | 1 | 1 | 1 | 1 |
| minValue | 26 | 147 | 27 | 86 | 87 | 85 | 72 | 35 | 193 | 15 | 129 |
| meanValue | 139 | 453 | 79.1 | 296 | 425 | 341 | 353 | 129 | 383 | 107 | 521 |
| stdValue | 26.3 | 76.6 | 14.4 | 57.4 | 88.8 | 57.4 | 77.6 | 29.1 | 41 | 18.5 | 131 |
| maxValue | 237 | 628 | 120 | 410 | 624 | 467 | 531 | 184 | 439 | 150 | 735 |
| HISTO_Skewness | -0.388 | -1.08 | -0.587 | -0.505 | -0.91 | -1.02 | -0.613 | -0.522 | -1.64 | -1.37 | -0.82 |
| HISTO_Kurtosis | 3.38 | 4.59 | 3.66 | 2.63 | 3.88 | 4.78 | 3.2 | 2.58 | 6.97 | 5.46 | 2.95 |
| HISTO_Entropy_log2 | 5.01 | 5.21 | 5.2 | 5.44 | 5.31 | 5.15 | 5.35 | 5.49 | 4.62 | 4.93 | 5.56 |
| GLCM_Homogeneity | 0.299 | 0.224 | 0.24 | 0.229 | 0.231 | 0.229 | 0.24 | 0.187 | 0.194 | 0.282 | 0.198 |
| GLCM_Energy | 0.00237 | 0.00234 | 0.00216 | 0.00129 | 0.00177 | 0.00204 | 0.00219 | 0.00216 | 0.0173 | 0.00341 | 0.00266 |
| GLCM_Contrast | 49.4 | 121 | 95 | 98.8 | 107 | 103 | 119 | 200 | 160 | 83.5 | 236 |
| GLCM_Correlation | 0.569 | 0.241 | 0.396 | 0.616 | 0.355 | 0.307 | 0.4 | 0.25 | 0.0825 | 0.286 | 0.272 |
| GLCM_Entropy_log2 | 9.4 | 9.16 | 9.47 | 9.98 | 9.61 | 9.3 | 9.16 | 9.09 | 6.02 | 8.96 | 8.81 |
| GLCM_Dissimilarity | 5.08 | 8 | 7.2 | 7.49 | 7.61 | 7.52 | 7.82 | 10.8 | 9.7 | 6.23 | 11.2 |
| GLRLM_SRE | 0.945 | 0.972 | 0.964 | 0.97 | 0.97 | 0.97 | 0.967 | 0.98 | 0.985 | 0.955 | 0.975 |
| GLRLM_LRE | 1.27 | 1.12 | 1.16 | 1.14 | 1.13 | 1.13 | 1.15 | 1.09 | 1.06 | 1.21 | 1.11 |
| GLRLM_LGRE | 0.00126 | 0.00218 | 0.00324 | 0.00134 | 0.00169 | 0.00295 | 0.00231 | 0.00252 | 0.0105 | 0.00138 | 0.00591 |
| GLRLM_HGRE | 1260 | 1800 | 1410 | 1890 | 1770 | 1970 | 1670 | 1830 | 2600 | 2010 | 1930 |
| GLRLM_SRLGE | 0.0012 | 0.00216 | 0.0032 | 0.00131 | 0.00167 | 0.00293 | 0.00229 | 0.00251 | 0.0105 | 0.00136 | 0.0059 |
| GLRLM_SRHGE | 1190 | 1740 | 1360 | 1820 | 1710 | 1910 | 1610 | 1780 | 2560 | 1910 | 1860 |
| GLRLM_LRLGE | 0.0015 | 0.00228 | 0.00337 | 0.00143 | 0.00178 | 0.00302 | 0.0024 | 0.00257 | 0.0105 | 0.00148 | 0.00596 |
| GLRLM_LRHGE | 1630 | 2030 | 1660 | 2170 | 2040 | 2270 | 1980 | 2030 | 2790 | 2480 | 2210 |
| GLRLM_GLNU | 2220 | 38.3 | 86.1 | 63.7 | 81.9 | 47.1 | 24.3 | 17.1 | 4.98 | 125 | 12.9 |
| GLRLM_RLNU | 52800 | 1050 | 2280 | 2290 | 2500 | 1330 | 805 | 646 | 96.3 | 2630 | 484 |
| GLRLM_RP | 0.926 | 0.963 | 0.951 | 0.959 | 0.96 | 0.96 | 0.955 | 0.973 | 0.98 | 0.939 | 0.967 |
| NGLDM_Coarseness | 0.000136 | 0.00626 | 0.00289 | 0.00356 | 0.00278 | 0.00481 | 0.00845 | 0.00847 | 0 | 0.00227 | 0.012 |
| NGLDM_Contrast | 0.11 | 0.31 | 0.24 | 0.342 | 0.298 | 0.285 | 0.37 | 0.712 | 0 | 0.206 | 0.786 |
| NGLDM_Busyness | 2.51 | 0.052 | 0.121 | 0.117 | 0.116 | 0.0775 | 0.0498 | 0.0551 | 0 | 0.138 | 0.0303 |
| GLZLM_SZE | 0.672 | 0.776 | 0.756 | 0.723 | 0.764 | 0.753 | 0.773 | 0.815 | 0.849 | 0.749 | 0.843 |
| GLZLM_LZE | 73.3 | 5.58 | 12 | 5.46 | 5.69 | 5.99 | 6.17 | 3.26 | 2.05 | 21.7 | 4.22 |
| GLZLM_LGZE | 0.0016 | 0.00293 | 0.00494 | 0.00173 | 0.00228 | 0.00454 | 0.00334 | 0.00326 | 0.013 | 0.00221 | 0.00811 |

| Patient | 232 | 233 | 234 | 235 | 236 | 237 | 238 | 239 | 240 | 241 | 242 |
| --- | --- | --- | --- | --- | --- | --- | --- | --- | --- | --- | --- |
| Lesion (benign=0,malignant=1) | 1 | 1 | 1 | 1 | 1 | 1 | 1 | 1 | 1 | 1 | 1 |
| minValue | 54 | 170 | 42 | 32 | 158 | 55 | 98 | 34 | 155 | 19 | 30 |
| meanValue | 183 | 420 | 196 | 214 | 364 | 169 | 189 | 63.1 | 437 | 117 | 112 |
| stdValue | 29.5 | 67.7 | 34.4 | 72 | 50.3 | 35.9 | 22.4 | 6.84 | 75.6 | 27.6 | 19.7 |
| maxValue | 256 | 568 | 286 | 463 | 498 | 267 | 262 | 82 | 654 | 267 | 160 |
| HISTO_Skewness | -0.92 | -0.781 | -0.917 | 0.151 | -0.454 | -0.651 | -0.412 | -0.669 | -0.0363 | 0.678 | -0.868 |
| HISTO_Kurtosis | 4.14 | 3.44 | 4.05 | 2.74 | 3.34 | 2.85 | 3.56 | 4.64 | 3.51 | 4.79 | 3.8 |
| HISTO_Entropy_log2 | 5.14 | 5.36 | 5.09 | 5.37 | 5.25 | 5.35 | 5.1 | 4.71 | 5.21 | 4.79 | 5.2 |
| GLCM_Homogeneity | 0.235 | 0.2 | 0.243 | 0.241 | 0.224 | 0.223 | 0.23 | 0.222 | 0.217 | 0.383 | 0.269 |
| GLCM_Energy | 0.00212 | 0.002 | 0.00199 | 0.00152 | 0.00154 | 0.00167 | 0.00235 | 0.00344 | 0.00413 | 0.00478 | 0.00211 |
| GLCM_Contrast | 109 | 166 | 97.4 | 108 | 112 | 123 | 79.7 | 134 | 110 | 23.8 | 79.8 |
| GLCM_Correlation | 0.194 | 0.149 | 0.274 | 0.547 | 0.313 | 0.432 | 0.313 | 0.124 | 0.318 | 0.729 | 0.483 |
| GLCM_Entropy_log2 | 9.49 | 9.32 | 9.5 | 9.84 | 9.74 | 9.77 | 9.09 | 8.61 | 8.12 | 8.64 | 9.6 |
| GLCM_Dissimilarity | 7.6 | 9.65 | 7.22 | 7.72 | 7.88 | 8.16 | 6.94 | 8.52 | 8.02 | 3.38 | 6.34 |
| GLRLM_SRE | 0.968 | 0.977 | 0.967 | 0.963 | 0.971 | 0.969 | 0.974 | 0.965 | 0.982 | 0.916 | 0.957 |
| GLRLM_LRE | 1.14 | 1.1 | 1.15 | 1.18 | 1.12 | 1.14 | 1.12 | 1.16 | 1.08 | 1.45 | 1.2 |
| GLRLM_LGRE | 0.00198 | 0.00184 | 0.0017 | 0.00322 | 0.00142 | 0.00317 | 0.00216 | 0.00236 | 0.00356 | 0.00225 | 0.00135 |
| GLRLM_HGRE | 1780 | 1760 | 1730 | 872 | 1630 | 1330 | 1380 | 1640 | 1430 | 725 | 1760 |
| GLRLM_SRLGE | 0.00196 | 0.00183 | 0.00167 | 0.00312 | 0.0014 | 0.00314 | 0.0021 | 0.00234 | 0.00354 | 0.0021 | 0.00132 |
| GLRLM_SRHGE | 1720 | 1720 | 1670 | 842 | 1580 | 1280 | 1340 | 1590 | 1410 | 667 | 1670 |
| GLRLM_LRLGE | 0.00206 | 0.0019 | 0.0018 | 0.00375 | 0.0015 | 0.00333 | 0.0024 | 0.00247 | 0.00362 | 0.00303 | 0.00146 |
| GLRLM_LRHGE | 2060 | 1970 | 2040 | 1020 | 1850 | 1540 | 1540 | 1900 | 1540 | 1030 | 2160 |
| GLRLM_GLNU | 128 | 37 | 151 | 69.5 | 79.8 | 70.6 | 62.5 | 44.1 | 15.2 | 904 | 155 |
| GLRLM_RLNU | 3400 | 1210 | 4000 | 2270 | 2410 | 2280 | 1670 | 848 | 461 | 16200 | 4220 |
| GLRLM_RP | 0.957 | 0.969 | 0.955 | 0.95 | 0.962 | 0.959 | 0.965 | 0.954 | 0.976 | 0.888 | 0.942 |
| NGLDM_Coarseness | 0.00183 | 0.00498 | 0.00155 | 0.00269 | 0.00277 | 0.00281 | 0.00448 | 0.00518 | 0.0158 | 0.000478 | 0.00173 |
| NGLDM_Contrast | 0.262 | 0.42 | 0.225 | 0.337 | 0.253 | 0.371 | 0.185 | 0.263 | 0.312 | 0.064 | 0.22 |
| NGLDM_Busyness | 0.18 | 0.0773 | 0.201 | 0.223 | 0.131 | 0.141 | 0.0963 | 0.118 | 0.0468 | 0.964 | 0.19 |
| GLZLM_SZE | 0.764 | 0.793 | 0.75 | 0.726 | 0.753 | 0.747 | 0.739 | 0.729 | 0.829 | 0.68 | 0.737 |
| GLZLM_LZE | 8.69 | 3.59 | 7.58 | 9.02 | 5.13 | 9.07 | 4.06 | 7.14 | 2.78 | 785 | 18.5 |
| GLZLM_LGZE | 0.0029 | 0.00237 | 0.00237 | 0.00369 | 0.00184 | 0.00445 | 0.00204 | 0.00363 | 0.00447 | 0.0033 | 0.00206 |

| Patient | 243 | 244 | 245 | 246 | 247 | 248 | 249 | 250 | 251 | 252 | 253 |
| --- | --- | --- | --- | --- | --- | --- | --- | --- | --- | --- | --- |
| Lesion (benign=0,malignant=1) | 1 | 1 | 1 | 1 | 1 | 1 | 1 | 1 | 1 | 1 | 1 |
| minValue | 117 | 81 | 29 | 141 | 15 | 13 | 25 | 10 | 14 | 123 | 25 |
| meanValue | 339 | 303 | 139 | 258 | 75.5 | 77.6 | 206 | 54.6 | 101 | 328 | 108 |
| stdValue | 42.3 | 62.1 | 21.1 | 32.9 | 14.7 | 13.7 | 26.9 | 8.33 | 26.6 | 46.6 | 21.9 |
| maxValue | 481 | 448 | 182 | 397 | 113 | 108 | 263 | 76 | 160 | 446 | 149 |
| HISTO_Skewness | -1.13 | -0.633 | -1.4 | 0.282 | -1.08 | -1.07 | -1.2 | -1.52 | -0.528 | -0.57 | -0.969 |
| HISTO_Kurtosis | 5.5 | 3.18 | 5.41 | 4.22 | 4.05 | 5.05 | 6.11 | 6.44 | 2.94 | 3.38 | 3.66 |
| HISTO_Entropy_log2 | 4.77 | 5.38 | 4.9 | 5.01 | 5.04 | 5.05 | 4.72 | 4.84 | 5.46 | 5.18 | 5.31 |
| GLCM_Homogeneity | 0.308 | 0.203 | 0.287 | 0.277 | 0.23 | 0.254 | 0.29 | 0.28 | 0.183 | 0.271 | 0.197 |
| GLCM_Energy | 0.00384 | 0.00174 | 0.00374 | 0.00273 | 0.00264 | 0.00264 | 0.00404 | 0.00336 | 0.00169 | 0.00194 | 0.0028 |
| GLCM_Contrast | 53.7 | 158 | 76 | 55.7 | 159 | 72.5 | 54.2 | 71.2 | 199 | 72.9 | 144 |
| GLCM_Correlation | 0.381 | 0.192 | 0.313 | 0.54 | 0.215 | 0.43 | 0.326 | 0.267 | 0.392 | 0.526 | 0.313 |
| GLCM_Entropy_log2 | 8.83 | 9.56 | 8.94 | 9.14 | 9.06 | 9.06 | 8.57 | 8.8 | 9.42 | 9.52 | 8.74 |
| GLCM_Dissimilarity | 5.07 | 9.36 | 5.95 | 5.53 | 8.82 | 6.36 | 5.41 | 5.85 | 10.7 | 6.06 | 9.13 |
| GLRLM_SRE | 0.944 | 0.976 | 0.952 | 0.962 | 0.969 | 0.965 | 0.958 | 0.96 | 0.981 | 0.957 | 0.981 |
| GLRLM_LRE | 1.27 | 1.11 | 1.23 | 1.18 | 1.14 | 1.15 | 1.2 | 1.19 | 1.08 | 1.2 | 1.08 |
| GLRLM_LGRE | 0.00121 | 0.00177 | 0.000877 | 0.00233 | 0.00184 | 0.00151 | 0.000966 | 0.00161 | 0.00328 | 0.00107 | 0.00266 |
| GLRLM_HGRE | 1610 | 1650 | 2210 | 956 | 1680 | 2010 | 2460 | 1960 | 1640 | 1770 | 1980 |
| GLRLM_SRLGE | 0.00117 | 0.00175 | 0.000856 | 0.00228 | 0.00182 | 0.00149 | 0.00095 | 0.00159 | 0.00319 | 0.00104 | 0.00265 |
| GLRLM_SRHGE | 1510 | 1600 | 2100 | 921 | 1620 | 1940 | 2350 | 1880 | 1610 | 1690 | 1940 |
| GLRLM_LRLGE | 0.00138 | 0.00184 | 0.000976 | 0.00257 | 0.00193 | 0.00159 | 0.00104 | 0.0017 | 0.00362 | 0.00119 | 0.00269 |
| GLRLM_LRHGE | 2070 | 1850 | 2780 | 1120 | 1960 | 2350 | 3010 | 2360 | 1780 | 2140 | 2170 |
| GLRLM_GLNU | 266 | 44.4 | 220 | 108 | 51.3 | 61.7 | 94.6 | 109 | 25.4 | 114 | 19.5 |
| GLRLM_RLNU | 4920 | 1490 | 4490 | 2500 | 1230 | 1490 | 1850 | 2240 | 910 | 3210 | 599 |
| GLRLM_RP | 0.925 | 0.968 | 0.935 | 0.949 | 0.959 | 0.954 | 0.944 | 0.946 | 0.975 | 0.943 | 0.975 |
| NGLDM_Coarseness | 0.00129 | 0.00409 | 0.00137 | 0.0031 | 0.0041 | 0.00441 | 0.00364 | 0.00284 | 0.00616 | 0.00249 | 0.0103 |
| NGLDM_Contrast | 0.11 | 0.414 | 0.184 | 0.129 | 0.33 | 0.202 | 0.139 | 0.158 | 0.516 | 0.2 | 0.457 |
| NGLDM_Busyness | 0.229 | 0.0925 | 0.197 | 0.146 | 0.0859 | 0.0723 | 0.117 | 0.102 | 0.0699 | 0.169 | 0.0368 |
| GLZLM_SZE | 0.69 | 0.787 | 0.736 | 0.694 | 0.767 | 0.752 | 0.697 | 0.758 | 0.8 | 0.676 | 0.812 |
| GLZLM_LZE | 57 | 3.83 | 51.3 | 10.8 | 5.72 | 9.56 | 17.3 | 16.3 | 2.94 | 14.1 | 2.8 |
| GLZLM_LGZE | 0.00199 | 0.00228 | 0.00134 | 0.00317 | 0.00256 | 0.00216 | 0.0015 | 0.00253 | 0.00272 | 0.00145 | 0.00339 |

| Patient | 254 | 255 | 256 | 257 | 258 | 259 | 260 | 261 | 262 | 263 | 264 |
| --- | --- | --- | --- | --- | --- | --- | --- | --- | --- | --- | --- |
| Lesion (benign=0,malignant=1) | 1 | 1 | 1 | 1 | 1 | 1 | 1 | 1 | 1 | 1 | 1 |
| minValue | 19 | 44 | 232 | 90.4 | 169 | 322 | 106 | 65.3 | 48.3 | 21 | 201 |
| meanValue | 95.1 | 112 | 682 | 162 | 287 | 506 | 216 | 322 | 139 | 157 | 367 |
| stdValue | 13.7 | 15.5 | 126 | 14.2 | 41.1 | 38.2 | 27 | 146 | 15.7 | 21.3 | 57.1 |
| maxValue | 120 | 148 | 935 | 214 | 357 | 615 | 284 | 661 | 177 | 223 | 488 |
| HISTO_Skewness | -1.91 | -0.78 | -0.742 | -0.736 | -0.597 | -0.82 | -0.959 | 0.0656 | -1.05 | -0.994 | -0.659 |
| HISTO_Kurtosis | 7.87 | 4.4 | 3.43 | 4.26 | 2.6 | 4.34 | 4.1 | 1.57 | 5.25 | 7.11 | 3.43 |
| HISTO_Entropy_log2 | 4.67 | 5.1 | 5.4 | 4.86 | 5.62 | 4.99 | 5.15 | 5.59 | 4.9 | 4.66 | 5.23 |
| GLCM_Homogeneity | 0.316 | 0.229 | 0.2 | 0.3 | 0.145 | 0.261 | 0.25 | 0.245 | 0.279 | 0.307 | 0.176 |
| GLCM_Energy | 0.00624 | 0.00312 | 0.00241 | 0.00283 | 0.00316 | 0.00254 | 0.00285 | 0.00209 | 0.00251 | 0.004 | 0.0149 |
| GLCM_Contrast | 79.5 | 102 | 154 | 40.3 | 427 | 69.4 | 88.2 | 198 | 59.8 | 34.9 | 238 |
| GLCM_Correlation | 0.26 | 0.263 | 0.233 | 0.554 | -0.0564 | 0.407 | 0.402 | 0.605 | 0.439 | 0.413 | 0.339 |
| GLCM_Entropy_log2 | 8.22 | 8.75 | 8.96 | 9.03 | 8.47 | 9.11 | 8.87 | 10.3 | 9.17 | 8.42 | 6.26 |
| GLCM_Dissimilarity | 5.73 | 7.59 | 9.31 | 4.74 | 15.8 | 6.18 | 6.84 | 9.89 | 5.59 | 4.45 | 11.9 |
| GLRLM_SRE | 0.944 | 0.973 | 0.979 | 0.951 | 0.986 | 0.964 | 0.968 | 0.953 | 0.96 | 0.954 | 0.983 |
| GLRLM_LRE | 1.3 | 1.12 | 1.09 | 1.23 | 1.06 | 1.16 | 1.15 | 1.28 | 1.19 | 1.21 | 1.07 |
| GLRLM_LGRE | 0.00132 | 0.00212 | 0.00429 | 0.00108 | 0.00386 | 0.00181 | 0.00216 | 0.00551 | 0.000892 | 0.00118 | 0.0127 |
| GLRLM_HGRE | 2420 | 1900 | 1840 | 1450 | 1840 | 1710 | 1680 | 1060 | 2120 | 1950 | 1570 |
| GLRLM_SRLGE | 0.00129 | 0.0021 | 0.00428 | 0.00105 | 0.00385 | 0.00179 | 0.00214 | 0.00506 | 0.00087 | 0.00116 | 0.0127 |
| GLRLM_SRHGE | 2270 | 1840 | 1800 | 1370 | 1810 | 1640 | 1620 | 1030 | 2030 | 1860 | 1540 |
| GLRLM_LRLGE | 0.00143 | 0.00218 | 0.00434 | 0.00125 | 0.00391 | 0.00191 | 0.00225 | 0.00845 | 0.000996 | 0.00129 | 0.0128 |
| GLRLM_LRHGE | 3220 | 2140 | 2050 | 1800 | 1970 | 2020 | 1960 | 1240 | 2540 | 2360 | 1700 |
| GLRLM_GLNU | 82.7 | 30.6 | 17.9 | 318 | 12.6 | 80.9 | 34.6 | 113 | 190 | 91.6 | 3.51 |
| GLRLM_RLNU | 1370 | 773 | 607 | 6750 | 514 | 1950 | 928 | 4490 | 4290 | 1690 | 107 |
| GLRLM_RP | 0.922 | 0.964 | 0.971 | 0.935 | 0.981 | 0.952 | 0.957 | 0.932 | 0.946 | 0.939 | 0.978 |
| NGLDM_Coarseness | 0.00393 | 0.00877 | 0.0105 | 0.00112 | 0.00967 | 0.00365 | 0.00735 | 0.0012 | 0.00173 | 0.00402 | 0.0517 |
| NGLDM_Contrast | 0.196 | 0.282 | 0.475 | 0.092 | 0.915 | 0.172 | 0.269 | 0.899 | 0.12 | 0.105 | 0.976 |
| NGLDM_Busyness | 0.0708 | 0.0486 | 0.0377 | 0.293 | 0.0397 | 0.0933 | 0.0488 | 0.568 | 0.159 | 0.0821 | 0.0123 |
| GLZLM_SZE | 0.782 | 0.776 | 0.8 | 0.656 | 0.843 | 0.705 | 0.756 | 0.774 | 0.687 | 0.685 | 0.802 |
| GLZLM_LZE | 75.3 | 5.74 | 3.69 | 19.7 | 2.23 | 7.61 | 7.12 | 46.2 | 10.9 | 16.8 | 2.4 |
| GLZLM_LGZE | 0.00235 | 0.00298 | 0.00585 | 0.00148 | 0.00467 | 0.00275 | 0.00314 | 0.00376 | 0.00117 | 0.00189 | 0.0166 |

| Patient | 265 |
| --- | --- |
| Lesion (benign=0,malignant=1) | 1 |
| minValue | 50.3 |
| meanValue | 158 |
| stdValue | 22.3 |
| maxValue | 223 |
| HISTO_Skewness | -0.52 |
| HISTO_Kurtosis | 3.7 |
| HISTO_Entropy_log2 | 5.05 |
| GLCM_Homogeneity | 0.299 |
| GLCM_Energy | 0.00228 |
| GLCM_Contrast | 52.4 |
| GLCM_Correlation | 0.578 |
| GLCM_Entropy_log2 | 9.41 |
| GLCM_Dissimilarity | 5.2 |
| GLRLM_SRE | 0.947 |
| GLRLM_LRE | 1.27 |
| GLRLM_LGRE | 0.000855 |
| GLRLM_HGRE | 1690 |
| GLRLM_SRLGE | 0.000819 |
| GLRLM_SRHGE | 1590 |
| GLRLM_LRLGE | 0.00104 |
| GLRLM_LRHGE | 2160 |
| GLRLM_GLNU | 517 |
| GLRLM_RLNU | 12900 |
| GLRLM_RP | 0.928 |
| NGLDM_Coarseness | 0.000623 |
| NGLDM_Contrast | 0.123 |
| NGLDM_Busyness | 0.528 |
| GLZLM_SZE | 0.656 |
| GLZLM_LZE | 33.7 |
| GLZLM_LGZE | 0.00108 |
