# Supplementary material for: MRI-Based Machine Learning in Differentiation Between Benign and Malignant Breast Lesions
Source: Front Oncol. 2021 Oct 18;11:552634. doi: 10.3389/fonc.2021.552634 (PMC8558475; doi:10.3389/fonc.2021.552634)
Supplement: Supplementary file 3 [file Table_3.docx]

|  | Texture features selection methods | | | | |
| --- | --- | --- | --- | --- | --- |
| Texture features | DC | RF | LASSO | XGboost | GBDT |
| minValue | √ | √ | √ | √ | √ |
| meanValue |  |  |  |  | √ |
| stdValue |  | √ |  | √ | √ |
| maxValue |  |  | √ |  |  |
| HISTO_Skewness |  | √ |  |  | √ |
| HISTO_Kurtosis |  | √ |  |  |  |
| SHAPE_Volume (mL) |  | √ |  |  | √ |
| SHAPE_Volume (# vx) |  |  | √ |  |  |
| SHAPE_Sphericity | √ | √ |  | √ | √ |
| SHAPE_Compacity | √ | √ |  |  | √ |
| GLCM_Energy | √ |  |  |  | √ |
| GLCM_Contrast |  |  |  |  | √ |
| GLCM_Entropy_log10 | √ | √ |  |  |  |
| GLCM_Entropy_log2 | √ | √ |  |  | √ |
| GLRLM_HGRE |  |  | √ |  |  |
| GLRLM_SRHGE |  | √ |  |  |  |
| GLRLM_LRHGE |  | √ | √ | √ |  |
| NGLDM_Coarseness |  | √ |  |  |  |
| NGLDM_Contrast |  | √ |  |  | √ |
| NGLDM_Busyness |  | √ |  |  |  |
| GLZLM_LGZE |  | √ |  |  |  |
| GLZLM_HGZE | √ |  | √ |  | √ |
| GLZLM_SZLGE |  | √ |  |  | √ |
| GLZLM_SZHGE | √ | √ |  | √ |  |
| GLZLM_LZLGE |  | √ |  |  |  |
| GLZLM_LZHGE |  |  | √ |  | √ |
| GLZLM_ZLNU |  |  | √ |  | √ |

Supplementary table 3: The results of texture features selection
